# Direct Comparison of Cardiac Myosin-Binding Protein C With Cardiac Troponins for the Early Diagnosis of Acute Myocardial Infarction

**DOI:** 10.1161/CIRCULATIONAHA.117.028084

**Published:** 2017-10-16

**Authors:** Thomas E. Kaier, Raphael Twerenbold, Christian Puelacher, Jack Marjot, Nazia Imambaccus, Jasper Boeddinghaus, Thomas Nestelberger, Patrick Badertscher, Zaid Sabti, Maria Rubini Giménez, Karin Wildi, Petra Hillinger, Karin Grimm, Sarah Loeffel, Samyut Shrestha, Dayana Flores Widmer, Janosch Cupa, Nikola Kozhuharov, Òscar Miró, F. Javier Martín-Sánchez, Beata Morawiec, Katharina Rentsch, Jens Lohrmann, Wanda Kloos, Stefan Osswald, Tobias Reichlin, Ekkehard Weber, Michael Marber, Christian Mueller

**Affiliations:** From King’s College London BHF Centre, Rayne Institute, St Thomas’ Hospital, London, UK (T.K., J.M., N.I., M.M.); Department of Cardiology and Cardiovascular Research Institute Basel, University Hospital Basel, Switzerland (R.T., C.P., J.B., T.N., P.B., Z.S., M.R.G., K.W., P.H., K.G., S.L., S.S., D.F.W., J.C., N.K., J.L., W.K., S.O., T.R., C.M.); Department of General and Interventional Cardiology, University Heart Center Hamburg, Germany (R.T., M.R.G.); Emergency Department, Centre for Biomedical Network Research on Rare Diseases Instituto de Salud Carlos III, Hospital del Mar–IMIM, Barcelona, Spain (K.W.); Emergency Department, Hospital Clinic, Barcelona, Spain (O.M.); Global Research in Acute Conditions Network (O.M., F.J.M.S., B.M., C.M.); Emergency Department, Hospital Clinico San Carlos, Madrid, Spain (F.J.M.S.); 2nd Cardiology Department, Zabrze, University Silesia, Katowice, Poland (B.M.); Laboratory Medicine, University Hospital Basel, Switzerland (K.R.); and Institute of Physiological Chemistry, Martin Luther University Halle-Wittenberg, Germany (E.W.).

**Keywords:** cardiac myosin-binding protein C, cMyC, myocardial infarction, APACE, troponin I, troponin T

## Abstract

**Background:**

Cardiac myosin-binding protein C (cMyC) is a cardiac-restricted protein that is more abundant than cardiac troponins (cTn) and is released more rapidly after acute myocardial infarction (AMI). We evaluated cMyC as an adjunct or alternative to cTn in the early diagnosis of AMI.

**Methods:**

Unselected patients (N=1954) presenting to the emergency department with symptoms suggestive of AMI, concentrations of cMyC, and high-sensitivity (hs) and standard-sensitivity cTn were measured at presentation. The final diagnosis of AMI was independently adjudicated using all available clinical and biochemical information without knowledge of cMyC. The prognostic end point was long-term mortality.

**Results:**

Final diagnosis was AMI in 340 patients (17%). Concentrations of cMyC at presentation were significantly higher in those with versus without AMI (median, 237 ng/L versus 13 ng/L, *P*<0.001). Discriminatory power for AMI, as quantified by the area under the receiver-operating characteristic curve (AUC), was comparable for cMyC (AUC, 0.924), hs-cTnT (AUC, 0.927), and hs-cTnI (AUC, 0.922) and superior to cTnI measured by a contemporary sensitivity assay (AUC, 0.909). The combination of cMyC with hs-cTnT or standard-sensitivity cTnI (but not hs-cTnI) led to an increase in AUC to 0.931 (*P*<0.0001) and 0.926 (*P*=0.003), respectively. Use of cMyC more accurately classified patients with a single blood test into rule-out or rule-in categories: Net Reclassification Improvement +0.149 versus hs-cTnT, +0.235 versus hs-cTnI (*P*<0.001). In early presenters (chest pain <3 h), the improvement in rule-in/rule-out classification with cMyC was larger compared with hs-cTnT (Net Reclassification Improvement +0.256) and hs-cTnI (Net Reclassification Improvement +0.308; both *P*<0.001). Comparing the C statistics, cMyC was superior to hs-cTnI and standard sensitivity cTnI (*P*<0.05 for both) and similar to hs-cTnT at predicting death at 3 years.

**Conclusions:**

cMyC at presentation provides discriminatory power comparable to hs-cTnT and hs-cTnI in the diagnosis of AMI and may perform favorably in patients presenting early after symptom onset.

**Clinical Trial Registration:**

URL: https://www.clinicaltrials.gov. Unique identifier: NCT00470587.

## Abstract 2

Supplemental Digital Content is available in the text.

**Clinical Perspective**

**What Is New?**

- Cardiac myosin-binding protein C is a recently described novel biomarker of cardiac injury, and in small proof-of-concept studies its serum concentration rises and falls more rapidly than that of troponin T and I.
- This is the first study to assess the diagnostic and prognostic value of cardiac myosin-binding protein C in patients presenting with possible acute myocardial infarction.
- A rule-in/rule-out pathway using the novel biomarker was designed to compare discriminative power in a clinical setting.

**What Are the Clinical Implications?**

- Diagnostic accuracy of cardiac myosin-binding protein C for acute myocardial infarction was similar to that of high-sensitivity cardiac troponin T and high-sensitivity cardiac troponin I in the entire cohort but superior for those with chest pain of <3 hours (early presenters) when compared with high-sensitivity cardiac troponin T.
- Cardiac myosin-binding protein C has correctly triaged more patients to rule-out or rule-in groups than either high-sensitivity cardiac troponin I or high-sensitivity cardiac troponin T, leaving a much smaller proportion in the observation groups. This advantage may facilitate early discharge of low-risk patients.

Of the 130 million attendances to emergency departments (EDs) in the United States each year, ≈7 million (6%) are a result of acute chest pain.^1^ The assessment and triage of such patients has become increasingly complex because now only a small proportion of those with acute myocardial infarction (AMI) have the diagnostic ECG change of ST-segment elevation.^2^ Consequently, the identification of patients with AMI has become almost totally dependent on the measurement in the systemic circulation of cardiac troponin (cTn) I or cTnT. These biomarkers are released slowly.^3^ To overcome this hurdle, the analytic performance of the cTn assays has been enhanced markedly to measure the lower concentrations achieved before the late peak.^4^ Hence, the best assays can reliably measure cTn concentrations below the 99th percentile of the healthy population. These high-sensitivity (hs) assays are increasingly available and are the subject of national and international guidelines describing their use to achieve more rapid triage.^5,6^ In particular, the European guidelines recommend the use of assays for hs-cTnI and hs-cTnT to rapidly rule in and rule out AMI. Algorithms using widely spaced decision limits based on concentrations well below the population-defined 99th percentile (for rule-out) and above the 99th percentile (for rule-in) markedly improve the sensitivity of rule-out and specificity of rule-in. However, many patients presenting with chest pain have cTn concentrations that place them between these decision limits, in an indeterminate observation zone. These patients require repeat testing and subsequent second or third rounds of triage based on rates of change of cTn concentration over time.^6–8^ European guidelines also do not support the use of rapid rule-out/rule-in pathways using hs-cTn in patients presenting too early after chest pain onset—only after 3 hours is the rule-out threshold at the limit of detection guideline-compliant.^6^ This introduces systemic delays in allocation of evidence-based treatments and prolongs stay in the pressured and precious environment of the ED.

Originally discovered by Offer et al^9^ in 1973, the myosin-binding protein C family consists of 3 isoforms specific for slow skeletal, fast skeletal, and cardiac muscle, the latter being exclusively expressed in the heart from neonatal throughout human development.^10,11^ Among others,^12–15^ we have identified cardiac myosin-binding protein C (cMyC; Figure 1) as a new candidate biomarker of cardiac injury.^16^ In common with cTnT and cTnI, cMyC expression is restricted to the heart but is more abundant.^17^ Moreover, cMyC rises more rapidly in the systemic circulation than hs-cTnT after timed, iatrogenic AMI,^16^ perhaps as a result of its higher myocardial concentration.^18^ Using a recently developed hs assay for cMyC,^19^ a pilot study in 26 patients presenting early with AMI suggested that cMyC may rise more rapidly than hs-cTnI.^20^

**Figure 1.**
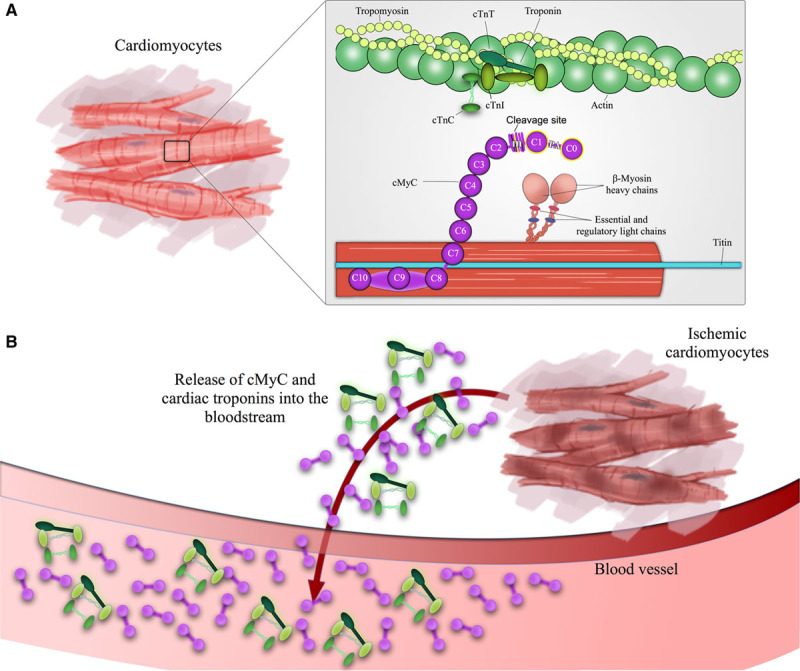
**Depiction of cardiac troponin and cardiac myosin-binding protein C release during myocardial injury.** Structure of cardiac myosin-binding protein C and cardiac troponins in (**A**) healthy cardiomyocytes and (**B**) ischemia-induced cardiomyocyte damage. The highlighted N-terminal domain C0C1 is the binding site for the previously developed monoclonal antibodies used for detection of the cardiac-specific isoform of cMyC.^16^ cMyC indicates cardiac myosin-binding protein C; cTnI, cardiac troponin I; and cTnT, cardiac troponin T.

The purpose of the current study is to compare the novel biomarker cMyC (measured on a research platform) against the most accurate currently available biochemical signals, hs-cTnI and hs-cTnT, for the early detection of AMI.

## Methods

### Study Design and Population

The APACE study (Advantageous Predictors of Acute Coronary Syndrome Evaluation) is an ongoing international multicenter diagnostic study (9 study centers in Switzerland, Spain, Poland, the Czech Republic, and Italy) designed to advance the early diagnosis of AMI.^4,21–23^ All patients >18 years of age presenting to the ED with acute chest discomfort possibly indicating AMI were eligible for recruitment if the onset of or peak chest pain symptoms were within the preceding 12 hours. Enrollment was independent of renal function, whereas patients with terminal kidney failure on chronic dialysis were excluded. For this analysis, the following patients were excluded (Figure I in the online-only Data Supplement): patients presenting with ST-segment elevation myocardial infarction, patients with missing levels of cMyC at presentation, and patients in whom the final diagnosis remained unclear after adjudication and at least 1 hs-cTnT level was elevated. This group is comprised of patients triaged and discharged after a negative gold-standard test at the time of enrollment (on a conventional cTn assay), who were later found to have an elevated hs-cTn result (Table I in the online-only Data Supplement). A proportion of patients had no levels of cMyC measured at presentation because of insufficient sample volume. Demographics of the patients excluded because of missing cMyC values, compared with those of the test cohort, appear in Table II in the online-only Data Supplement. The protocol for routine clinical assessment is also described in the online-only Data Supplement. To obtain follow-up data, patients were contacted 3, 12, 24, and 36 months after discharge via telephone, email, or letter. Additionally, information regarding death during follow-up was obtained from the patient’s hospital notes, the family physician’s records, and the national registry on mortality.

The study was carried out according to the principles of the Declaration of Helsinki and approved by the local ethics committees. Written informed consent was obtained from all patients. T.K., R.T., and C.M. had full access to all the data in the study and take responsibility for its integrity and the data analysis. The authors designed the study, gathered and analyzed the data according to the STARD guidelines (Standards for Reporting Diagnostic accuracy studies) for studies of diagnostic accuracy (Table III in the online-only Data Supplement), vouch for the data and analysis, wrote the paper, and decided to publish.

### Adjudicated Final Diagnosis

Adjudication of the final diagnosis was performed centrally according to the universal definition of MI, incorporating levels of hs-cTnT as the adjudicating biomarker.^24^ It was based on extensive patient documentation derived from 2 sets of data. First, clinical data were derived from routine clinical investigations, including all available medical records (eg, patient history, physical examination, results of laboratory testing including serial local hs-cTn, radiological testing, ECG, echocardiography, cardiac exercise stress test, lesion severity and morphology at coronary angiography) pertaining to the patient from the time of ED presentation to 90-day follow-up. Second, study-specific assessment was collected, including 34 chest pain characteristics and serial hs-cTnT measurements to take advantage of the higher sensitivity and higher overall diagnostic accuracy offered by the more sensitive assays, as previously published.^4,21^ In situations of disagreement about the diagnosis, cases were reviewed and adjudicated in conjunction with a third cardiologist. In brief, AMI was diagnosed when evidence indicated myocardial necrosis in association with a clinical setting consistent with myocardial ischemia. Myocardial necrosis was diagnosed by ≥1 (h)s-cTn value above the 99th percentile together with a significant rise or fall.^25–27^ All other patients were classified into the categories of unstable angina, cardiac but noncoronary disease (eg, tachyarrhythmias, perimyocarditis), noncardiac chest pain, and symptoms of unknown origin.

### Measurement of cMyC, hs-cTnI, hs-cTnT, and Standard-Sensitivity cTnI

Blood samples for the determination of cMyC, hs-cTnI, hs-cTnT, and standard-sensitivity (s) cTnI were collected into heparin plasma and serum tubes at presentation to the ED and serially thereafter (at time points 1 h, 2 h, 3 h, and 6 h). Serial sampling was discontinued when a diagnosis of AMI was certain and treatment required patient transfer to the coronary care unit or catheter laboratory. After centrifugation, samples were frozen at −80°C until they were assayed in a blinded fashion in a dedicated core laboratory. cMyC was measured using the previously established hs assay on the Erenna platform performed by Millipore Sigma.^19^ The assay has a limit of detection of 0.4 ng/L and a lower limit of quantification of 1.2 ng/L. The 99th percentile cutoff point determined previously (in patients without obstructive coronary artery disease on invasive angiography) is 87 ng/L.^19^ Details of the assays used for hs-cTnI, hs-cTnT, and s-cTnI are described in the online-only Data Supplement.

### Early Guideline-Based Triage and Net Reclassification Improvement

The European Society of Cardiology (ESC) has published a rapid rule-in/rule-out pathway in the 2015 non-ST-segment elevation MI guidelines using hs-cTn at 0 hours and 1 hour to risk-stratify patients into rule-out, observe, and rule-in categories.^6^ Such categorization did not drive clinical decisions in this cohort, but it was used to compare the potential clinical utilities of cMyC and hs-cTn as triage tools. For this purpose, we have compared the categorical discrimination of hs-cTnT, hs-cTnI, and cMyC at presentation only (without subsequent delta measurements). In brief, the ESC pathway classifies patients, based on the presentation sample at 0 hours, into rule-out with an hs-cTnT level <5 ng/L and hs-cTnI <2 ng/L and into rule-in (for both assays) at ≥52 ng/L.^6^ The ESC advocates the use of the pathway only in patients with ≥3 hours since chest pain onset; for completeness, we have presented results for all patients, <3 and ≥3 hours since chest pain onset alone.

For cMyC we separated the cohort into derivation and validation cohorts (a randomized 3:7 split; for comparison see Table IV in the online-only Data Supplement). The rule-out threshold was derived from a predefined sensitivity of ≥99.5% and rule-in from a predefined specificity >95% for the gold-standard diagnosis of AMI. This resulted in a rule-out threshold of ≤10 ng/L and a rule-in threshold of >120 ng/L for cMyC (Figure II in the online-only Data Supplement). These thresholds were then used in the validation cohorts to compare cMyC against both hs-cTnT and hs-cTnI. Net Reclassification Improvement (NRI) operates as follows. Each patient is first assigned a classification (rule-out, observe, or rule-in) based on cutoff values of hs-cTnI/hs-cTnT in the presentation blood sample (the initial model). The same cohort is then reclassified to the same 3 groups based on the cMyC cutoff values (the new model). This reclassification may correctly or incorrectly reallocate a patient (eg, a patient who went on to be diagnosed with an AMI may be correctly reclassified from observe to rule-in or incorrectly reclassified from observe to rule-out). The NRI analysis defines separate categorical NRI values for those patients who were ultimately diagnosed with AMI (NRI_AMI_) and those who were not (NRI_noAMI_; (range, −1 to +1). Dimensionless NRI reflects the unweighted net movement of all patients regardless of final diagnosis (range, −2 to +2). NRI_AMI_ is positive if there is a net movement of patients with adjudicated AMI into higher risk classifications using cMyC (the new model). NRI_noAMI_ is positive when a net movement of patients occurs without an adjudicated diagnosis of AMI into lower risk classifications using cMyC (the new model).^28^ NRI calculations were performed for the validation cohort, early presenters (<3 hours since onset of chest pain; ESC guideline not applicable), and late presenters (≥3 hours since onset; ESC guideline applicable). Tables are presented in full where appropriate.

### Statistical Analysis

All data are expressed as medians (first quartile, third quartile) or means (SD) for continuous variables (compared with the Mann-Whitney *U* test or Student *t* test) and for categorical variables as numbers and percentages (compared with Pearson χ^2^). Hypothesis testing was 2-tailed, and *P* values <0.05 were considered statistically significant. No adjustment for multiple comparisons was performed.

Discrimination power was quantified by the area under the receiver-operating characteristics curve (AUC) for each biomarker with all cases available, using 1000 stratified bootstrap replicates to calculate confidence intervals (CIs). Logistic regression was used to combine cMyC levels with hs-cTnT, hs-cTnI, or s-cTnI values for the assessment of an incremental value using 2 biomarkers at presentation. Subgroup analysis was performed for patients presenting early, defined as chest pain onset ≤3 hours of presentation to the ED. This is a particular limitation of the published ESC guidance on the use of hs-cTn for risk stratification because the rapid rule-out/rule-in algorithms are only applicable to patients with chest pain onset >3 hours.

Predictive value of the biomarkers during follow-up was assessed 2-fold: We calculated (1) Harrell’s C statistic for each biomarker at presentation for end points AMI, death or the composite of AMI, and all-cause mortality during follow-up (excluding the index event), and a higher C index indicates a higher probability of an event occurring during follow-up with higher biomarker values^29^; and (2) Kaplan-Meier survival curves. Cox regression analysis was performed as follows. All available biomarker levels were divided into quintiles and groups according to rule-out, observe, and rule-in classification. Unadjusted Cox proportional hazard regression models were fitted for 30-day and 3-year follow-up for each group with the lowest quintile (or risk group, respectively) normalized to a hazard ratio of 1 and assessed using the likelihood-ratio test. Cox coefficients and thus hazard ratios were not calculated if the lowest risk group did not suffer any events, which would invalidate the regression model. NRI statistics were calculated as categorical values.^28,30^ The integrated discrimination improvement values quoted reflect a category-free (positive or negative) change in model performance. CIs for cutoff thresholds, NRI, and integrated discrimination improvement statistics were derived using 1000 bootstrap replicates. All statistical analyses were performed using R, version 3.3.0 GUI 1.68 (The R Foundation for Statistical Computing), including packages ggplot2, R Markdown, RStudio, PredictAbel, survival, Hmisc, compare, and ROCR.

## Results

### Baseline Characteristics

A total of 1954 unselected patients eligible for this analysis were enrolled (Figure I in the online-only Data Supplement). Median age was 62 years, 31% were women, and 36% had a prior history of coronary artery disease (Table 1). Overall, 1469 patients (75%) had no significant electrocardiographic abnormalities at presentation to the ED. Median time since onset of chest pain was 5 hours (interquartile range [IQR], 3, 12), with a median of 3 hours (IQR 2, 7) since peak chest pain severity.

**Table 1.**
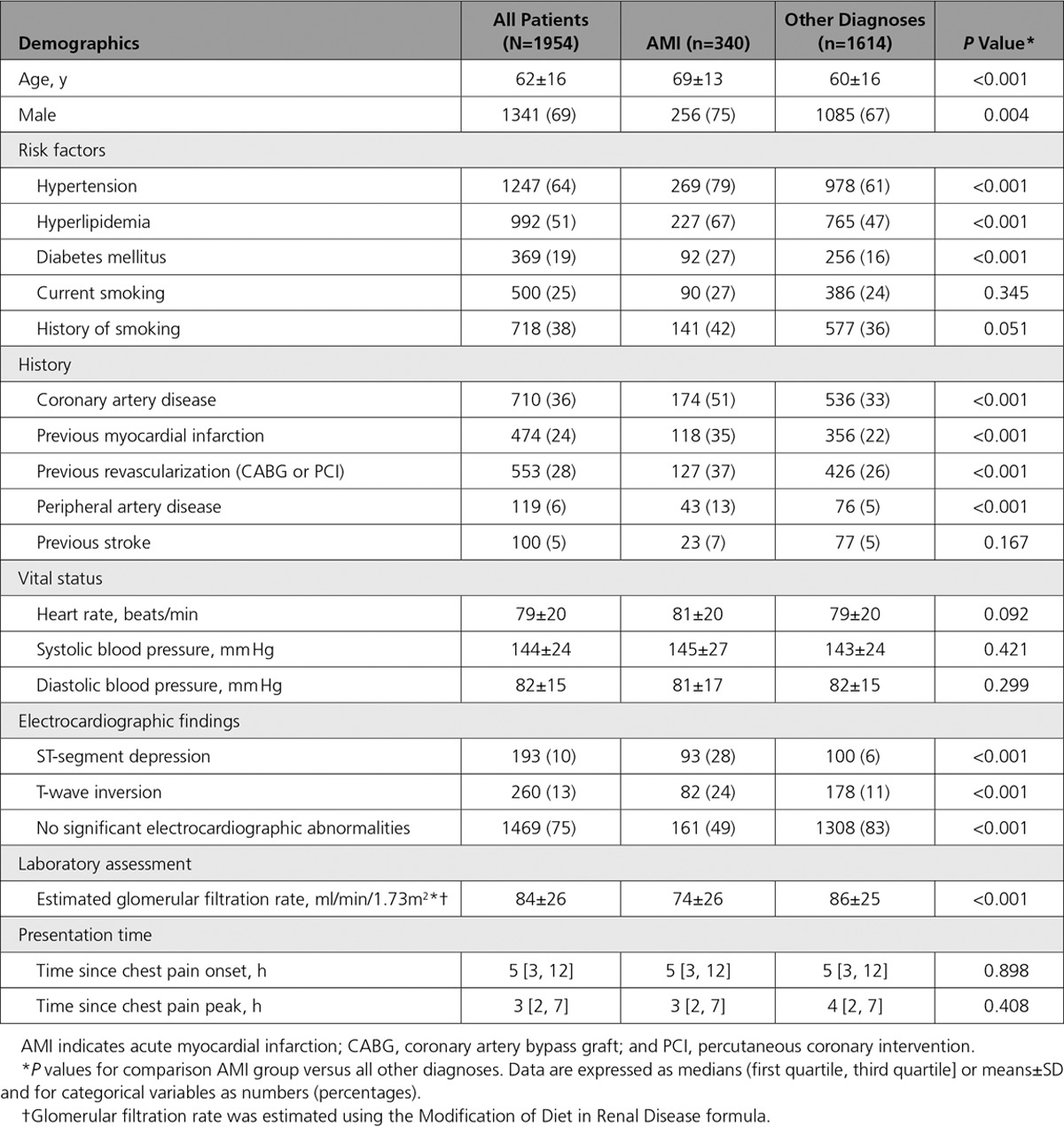
Demographics

The adjudicated final diagnosis was AMI in 340 (17%) patients, unstable angina in 10%, symptoms of cardiac origin other than coronary artery disease in 14%, noncardiac symptoms in 54%, and symptoms of unknown origin in 5%.

Median follow-up for the entire cohort was 772 days (IQR 731, 907); of those not sustaining any events in the monitoring period (AMI or death), the median follow-up was 792 days (IQR 738, 923). A total of 165 (8%) patients died during the 3-year follow-up; 1903 patients (97%) exceeded 90 days of follow-up; of those who did not (n=51, 3%), 27 (1%) sustained a cardiovascular death.

### Distribution of Biomarker Concentrations

**Figure 2.**
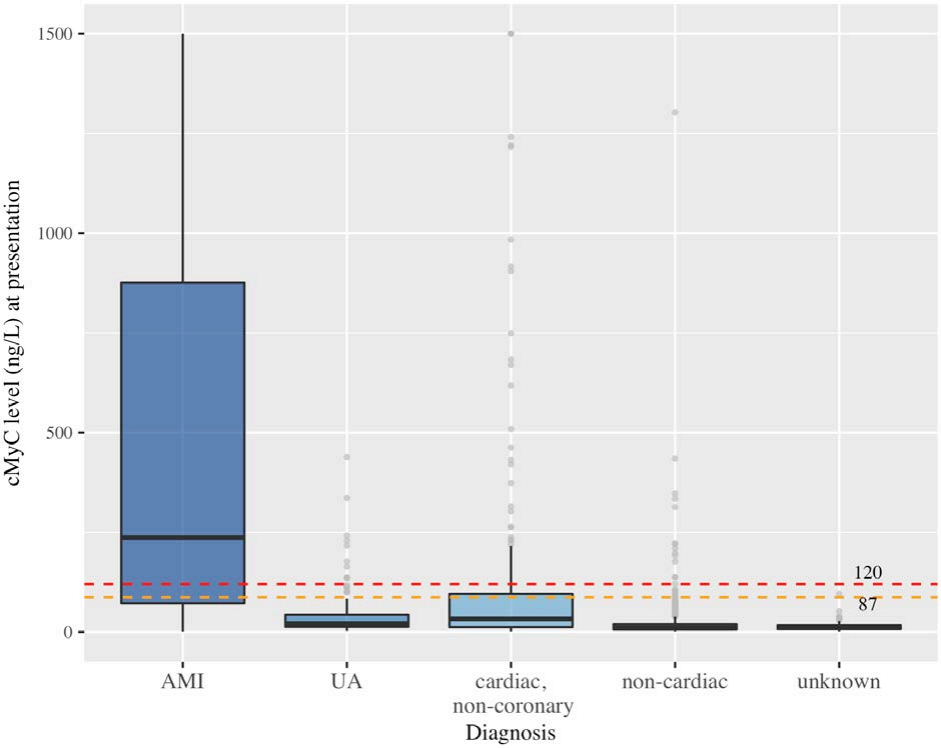
**Baseline distribution of cMyC levels at presentation to the emergency department in all patients based on adjudicated final diagnosis.** Boxes represent interquartile ranges (IQR). Whiskers extend to 1.5*IQR from the hinges (*y* axis capped at 1500 ng/L, outliers represented by light gray bullets); 87 ng/L represents the 99th percentile based on a previous study and 120 ng/L the cutoff threshold for diagnostic rule-in of AMI at presentation. AMI, median, 237 ng/L (IQR 71, 876 ng/L); unstable angina, median, 21 ng/L (IQR 13, 43 ng/L); cardiac symptoms of origin other than coronary artery disease, median, 33 ng/L (IQR 12, 96 ng/L); noncardiac symptoms, median, 10 ng/L (IQR 6, 19 ng/L; symptoms of unknown origin, median, 11 ng/L (IQR 7, 16 ng/L) (*P*<0.001 for all comparisons with patients with AMI). AMI indicates acute myocardial infarction; cMyC, cardiac myosin-binding protein C; and UA, unstable angina.

As shown in Figure 2, cMyC levels were significantly higher in patients with AMI (n=340) compared with patients with other diagnoses (AMI, median 237 ng/L [IQR 71, 876 ng/L]; unstable angina, median 21 ng/L [IQR 13, 43 ng/L]; cardiac symptoms of origin other than coronary artery disease, median 33 ng/L [IQR 12, 96 ng/L]; noncardiac symptoms, median 10 ng/L [IQR 6, 19 ng/L]; symptoms of unknown origin, median 11 ng/L [IQR 7, 16 ng/L]; *P*<0.001 for all comparisons with patients with AMI). Similarly, blood concentrations of hs-cTnT, hs-cTnI, and s-cTnI were significantly higher in AMI compared with other final diagnoses (median biomarker concentrations are displayed in Tables V and VI in the online-only Data Supplement). Overall, blood concentrations of cMyC in relation to the limit of detection were higher than those of hs-cTn in all diagnostic categories (Table V in the online-only Data Supplement). Noncardiac sources of cMyC variation were previously investigated in an ambulatory cohort.^19^ Results of comparison within the groups with AMI and noncardiac symptoms have been displayed in Tables VII and VIII in the online-only Data Supplement.

### Discrimination Power

**Figure 3.**
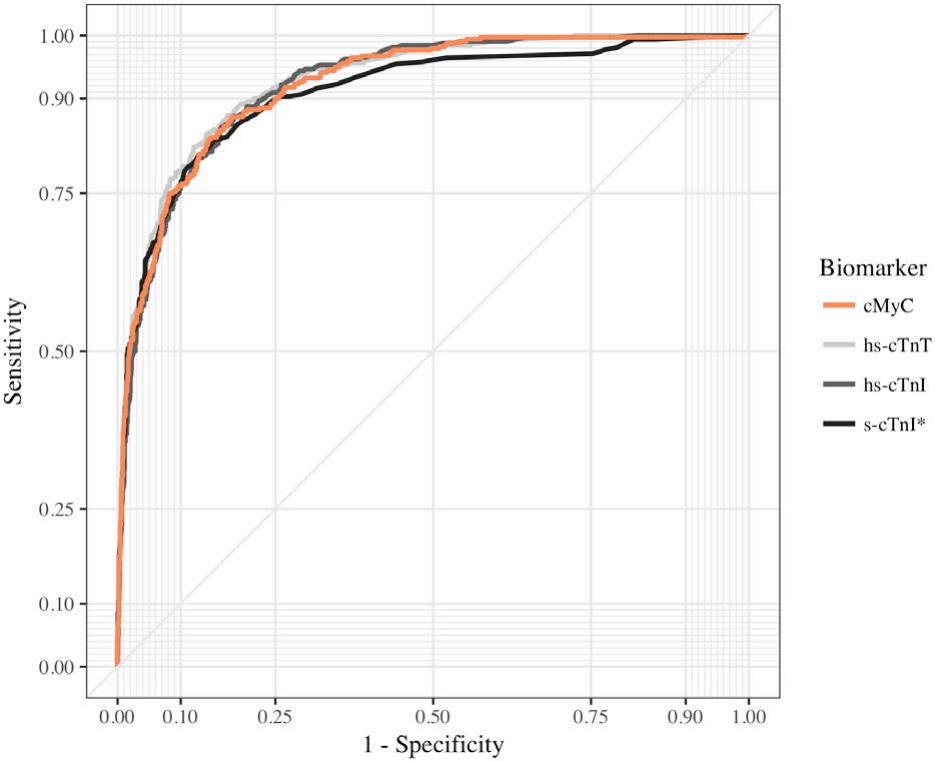
**Receiver operating characteristic** (**ROC) curves for individual biomarkers.** Diagnostic performance of cMyC, hs-cTnT, hs-cTnI, and s-cTnI in the early diagnosis of acute myocardial infarction (AMI) based on presentation blood sample and adjudicated AMI diagnosis. ROC curves describing the performance of cMyC (orange line; area under the curve [AUC], 0.924), hs-cTnT (light gray line; AUC, 0.927), hs-cTnI (dark gray line; AUC, 0.922), and s-cTnI (black line; AUC, 0.909*) (*P<0.05). cMyC indicates cardiac myosin-binding protein C; hs-cTnI, high-sensitivity cardiac troponin I; hs-cTnT, high-sensitivity cardiac troponin T; and s-cTnI, standard-sensitivity cardiac troponin I.

In blood drawn at presentation, the discrimination of cMyC for AMI, as quantified by the AUC, was 0.924 (95% CI, 0.910–0.939), compared to the AUCs for hs-cTnT 0.927 (95% CI, 0.913–0.941; *P*=0.573 for direct comparison), hs-cTnI 0.922 (95% CI, 0.908–0.936; *P*=0.993 for direct comparison), and s-cTnI 0.909 (95% CI, 0.889–0.928; *P*=0.024 for direct comparison) (Table 2, Figure 3).

**Table 2.**
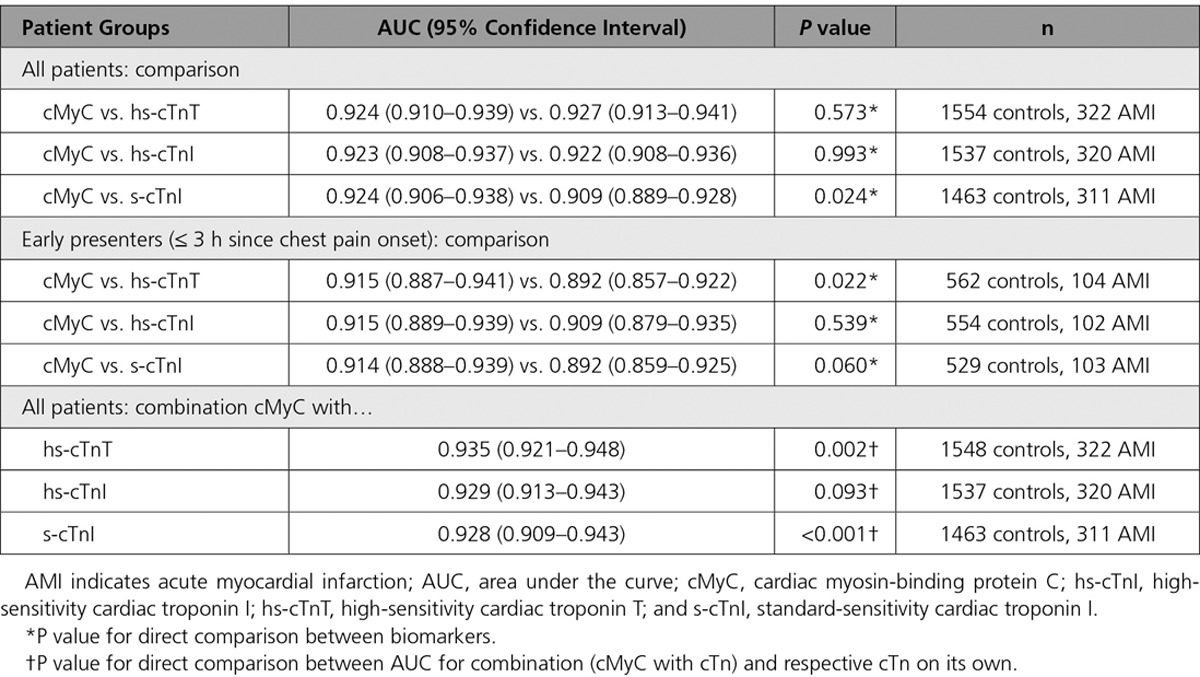
Area Under the Receiver-Operating Characteristics Curve: Comparisons Between Biomarkers

### Early Presenters

In patients presenting ≤3 hours of symptom onset (n=694, with AMI adjudicated in 16%), the AUC for cMyC was 0.915 (95% CI, 0.887–0.941), compared with the AUCs for hs-cTnT, 0.892 (95% CI, 0.857–0.922; *P*=0.022); hs-cTnI, 0.909 (95% CI, 0.879–0.935; *P*=0.539); and s-cTnI, 0.892 (95% CI, 0.859–0.925; *P*=0.060) (Table 2).

### Combination of cMyC With cTn

AUC for the combination of cMyC with hs-cTnT was 0.935 (95% CI, 0.921–0.948; *P*=0.002 for comparison with hs-cTnT alone); cMyC with hs-cTnI, 0.929 (95% CI, 0.913–0.943; *P*=0.093 for comparison with hs-cTnI alone), and cMyC with s-cTnI, 0.928 (95% CI, 0.909–0.943; *P*<0.001 for comparison with s-cTnI alone) (Table 2, Figure III in the online-only Data Supplement).

### Classification Function of Cutoff Values for Risk Groups

Sensitivity, specificity, and negative and positive predictive values were calculated for derivation (Tables IX and X in the online-only Data Supplement) and validation cohorts based on cutoffs published in the 2015 ESC guideline.^6^ In the validation cohort (n=1 368,233 events), hs-cTnT has a sensitivity of 99.6% (95% CI, 98.5−100) and negative predictive value of 99.7% (95% CI, 99−100) at the rule-out threshold of 5 ng/L, and a specificity of 97.1% (95% CI, 96.1−98) and positive predictive value of 80.1% (95% CI, 73.2–86.2) at the rule-in threshold (52 ng/L); and hs-cTnI has a sensitivity of 100% (95% CI, 100−100) and negative predictive value of 100% (95% CI, 100−100) at 2 ng/L, and a specificity of 94.5% (95% CI, 93–95.8) and positive predictive value of 70.4% (95% CI, 63.6–76.5) for rule-in (Tables 3 and 4). After obtaining clinically meaningful cutoff thresholds in the internal derivation cohort (based on sensitivity ≥99.5% and specificity >95%; Tables IX and X and Figure II in the online-only Data Supplement), these were tested in the validation cohort. At a threshold of 10 ng/L for rule-out, cMyC achieves a sensitivity of 99.6% (95% CI, 98.6−100) and negative predictive value of 99.8% (95% CI, 99.3−100). At 120 ng/L for the rule-in threshold, cMyC achieves a specificity of 94.7% (95% CI, 93.3–95.9) and positive predictive value of 71% (95% CI, 64.9–77.2) (all data are listed in Tables 3 and 4).

**Table 3.**
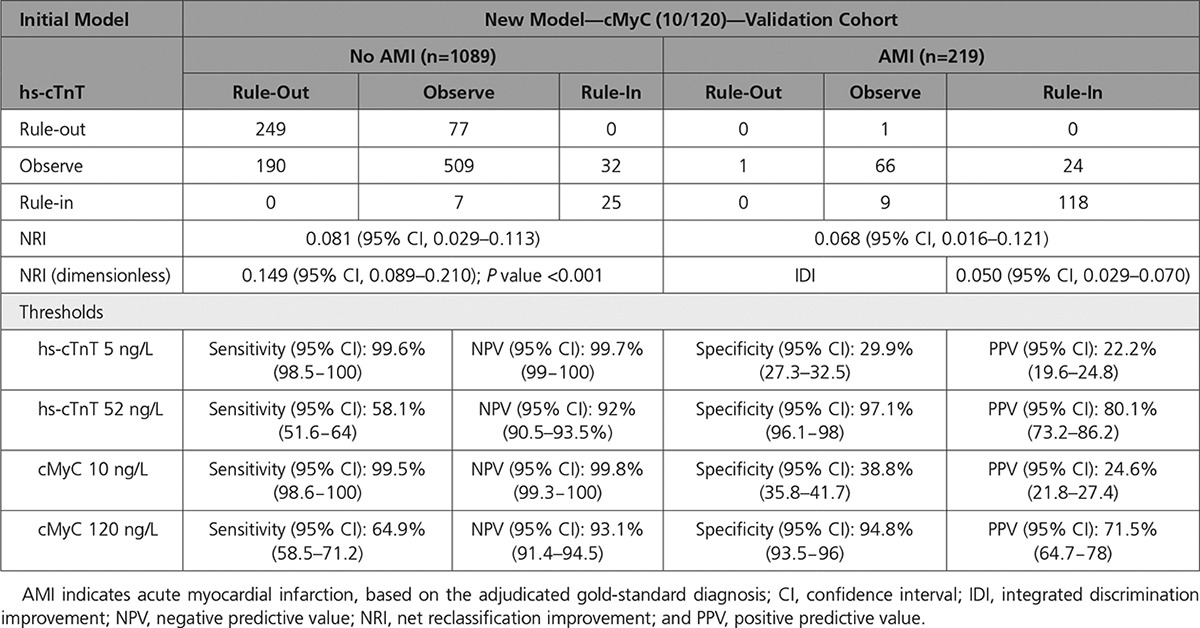
Net Reclassification Improvement: hs-cTnT (Validation Cohort)

**Table 4.**
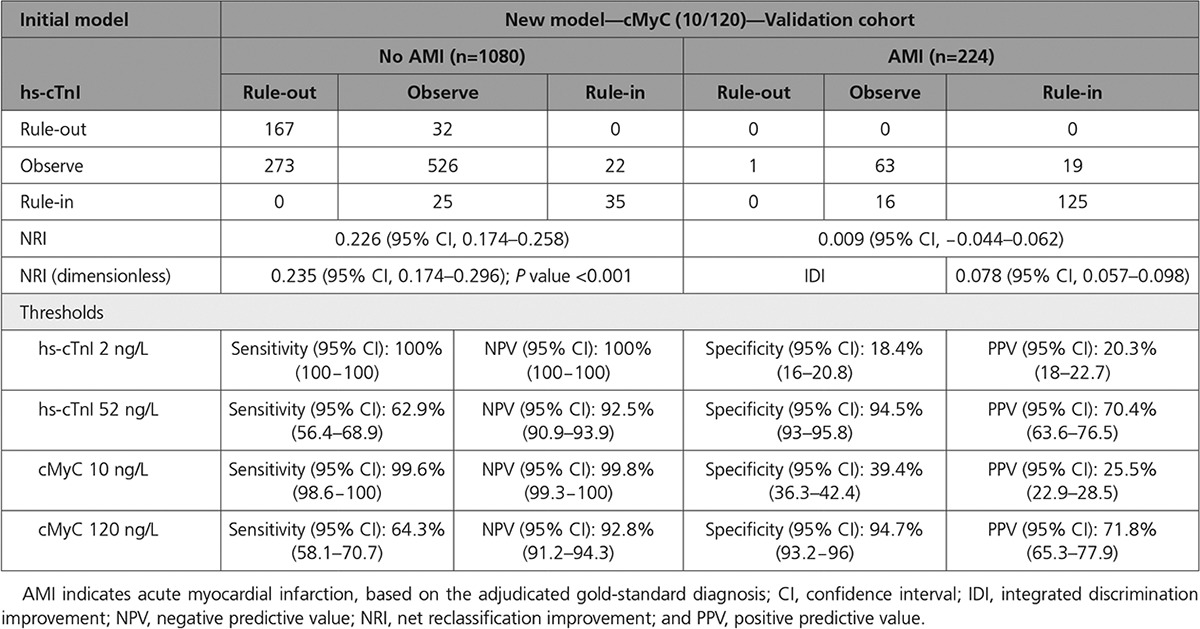
Net Reclassification Improvement: hs-cTnI (Validation Cohort)

All data for the groups of early (<3 hours of chest pain) and late presenters (≥3 hours of chest pain) are presented in Tables XI and XII in the online-only Data Supplement. In short, in early presenters, cMyC demonstrates higher sensitivity than hs-cTnT (100% versus 98.8%) and greater specificity (46.4% versus 33.3%) at the rule-out threshold (10 ng/L). Sensitivity is similar for cMyC and hs-cTnI, however, again with greater specificity for cMyC (47.1% versus 23.2%). In the group of late presenters, cMyC yields higher specificity (37.3% versus hs-cTnT, 28.4%; 38.1% versus hs-cTnI, 15.9%) at the rule-out threshold with otherwise comparable sensitivity. Specificity for adjudicated diagnosis of AMI was individually assessed at the 99th percentile in Table XIII in the online-only Data Supplement.

### Risk Group Reclassification

**Figure 4.**
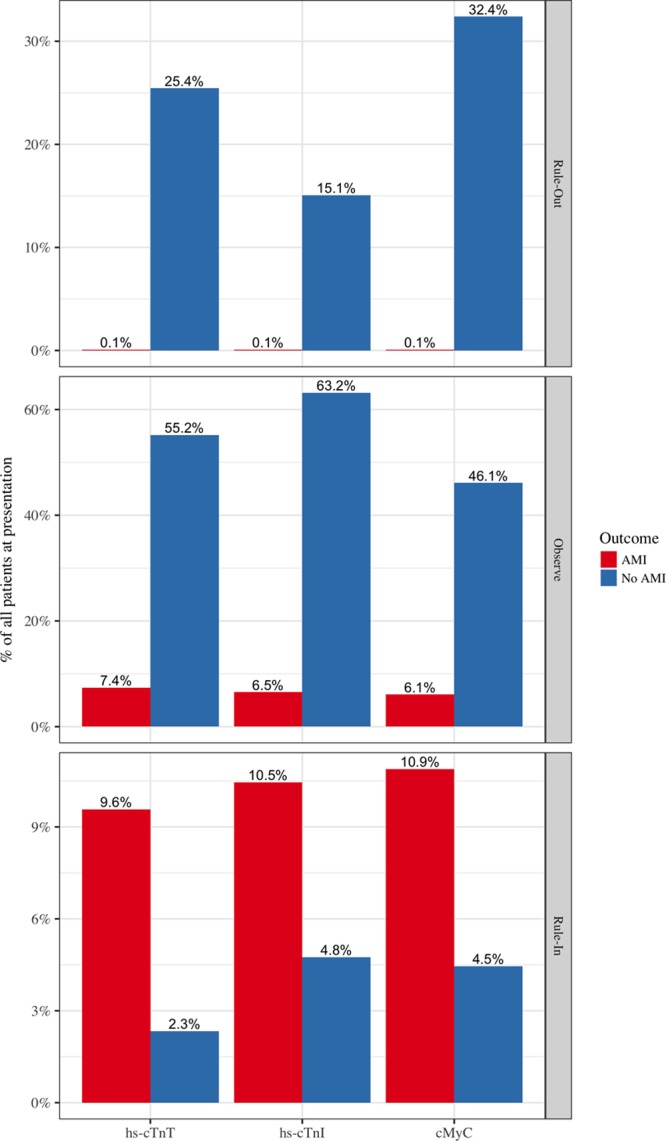
**Distribution of patients in different risk categories after presentation blood tests.** Data are based on European Society of Cardiology guideline 2015^6^ for hs-cTnT and hs-cTnI and newly developed cutoff thresholds for cMyC at ≤10 ng/L for rule-out and >120 ng/L for rule-in of myocardial infarction. AMI indicates acute myocardial infarction; cMyC, cardiac myosin-binding protein C; hs-cTnI, high-sensitivity cardiac troponin I; and hs-cTnT, high-sensitivity cardiac troponin T.

The distribution of patients in risk groups rule-out, observe, and rule-in based on the initial blood test (either hs-cTnT, hs-cTnI, or cMyC) is displayed in Figure 4 (validation cohort, n=1368, AMI in 17%). cMyC classified 443 patients (32.4%) safely as rule-out, compared with 348 (25.4%) with hs-cTnT and 206 (15.1%) with hs-cTnI, predominantly by reducing the size of the observation group.

In the validation cohort (Tables 3 and 4), cMyC at presentation was superior to hs-cTnT with NRI +0.149 (NRI_noAMI_ +0.081, NRI_AMI_ +0.068; *P*<0.001) and to hs-cTnI with NRI +23.5 (NRI_noAMI_ +0.226, NRI_AMI_ +0.009; *P*<0.001). In the cohort of early presenters (<3 hours of chest pain), cMyC was superior to hs-cTnT with NRI +0.256 (NRI_noAMI_ +0.256, NRI_AMI_ +0.128; *P*<0.001) and to hs-cTnI with NRI +0.308 (NRI_noAMI_ +0.257, NRI_AMI_ +0.051; *P*<0.001) (Table XI in the online-only Data Supplement). In the cohort of late presenters (≥3 hours of chest pain), cMyC was superior to hs-cTnT with NRI +0.133 (NRI_noAMI_ +0.084, NRI_AMI_ +0.049; *P*<0.001) and to hs-cTnI with NRI +0.227 (NRI_noAMI_ +0.240, NRI_AMI_ -0.012; *P*<0.001) (Table XII in the online-only Data Supplement).

### Prognostic Performance of cMyC

As quantified by Harrell’s C statistic calculated from the presentation sample (Table XIV in the online-only Data Supplement), cMyC matched the performance of hs-cTnT in predicting AMI (excluding index event), death, and the composite end point within a 3-year follow-up. Compared with hs-cTnI, there was a statistically different but numerically small improvement in predicting death and the composite end point at 3 years: cMyC C index 0.767 versus hs-cTnI 0.732 (*P*=0.001) and 0.746 versus 0.722 (*P*=0.008), respectively; AMI was comparable. cMyC was significantly better at predicting AMI, death, or the composite end point when compared with cTnI.

For the calculation of cumulative hazard ratios for all-cause mortality using a Cox regression model, each biomarker was separated into quintiles. The hazard ratios for hs-cTnT at 3-year follow-up were 2.3 (95% CI, 0.6–9.0) in the second quintile, 7.7 (95% CI, 2.3–25.8) in the third quintile, 17.7 (95% CI, 5.5–57.1) in the fourth quintile, and 33.6 (95% CI, 10.6–106.3) in the fifth quintile (*P*<0.05 for all except second quintile). The hazard ratios for hs-cTnI were 6.6 (95% CI, 1.5–29.2), 11.3 (95% CI, 2.7–48.3), 25.1 (95% CI, 6.1–103.3), and 39.7 (95% CI, 9.7–161.8), respectively (*P*<0.05 for all quintiles). The hazard ratios for cMyC were 2.6 (95% CI, 0.7–10.0), 7.8 (95% CI, 2.3–25.9), 17.2 (95% CI, 5.4–55.0), and 29.4 (95% CI, 9.3–93.2) (*P*<0.05 for all except second quintile). Survival curves for cMyC and hs-cTn assays are displayed in Figures IVA−C in the online-only Data Supplement for 3-year and 30-day follow-up.

## Discussion

To our knowledge, cMyC is the first cardiac-restricted protein to be analyzed as a diagnostic test for AMI since cTn. In this diagnostic multicenter study, we compared its diagnostic performance to cTnI and cTnT, measured using the leading hs assays recommended in current practice guidelines,^6^ in a well-characterized and large cohort of patients presenting with symptoms suggestive of AMI. Discrimination for MI with cMyC was similar to that of hs-cTnT and hs-cTnI and superior to s-cTnI. In patients presenting <3 hours of chest pain onset, cMyC was superior to hs-cTnT, despite the latter’s use as the adjudicating biomarker. Using cutoffs for cMyC calibrated against those recommended in the guidelines,^6^ cMyC correctly and safely rules out and rules in AMI in a greater proportion of patients than either hs-cTnT or hs-cTnI. These findings indicate that cMyC may be better able to triage patients presenting to the ED with suspected AMI.

cTnT and cTnI have transformed the management of patients with suspected AMI, and their importance is enshrined in the Universal Definition of Myocardial Infarction.^31^ Consequently, AMI events are identified/adjudicated based on a significant rise or fall in cTnT/I blood concentration. This definition has harmonized clinical care and clinical research but also has introduced an inherent bias in favor of cTnT/cTnI versus novel diagnostic biomarkers in studies such as ours. cMyC is not part of the troponin complex and has a distinct location within the cardiac sarcomere (Figure 1). For these reasons, our findings regarding the performance of cMyC against the hs-cTnT and hs-cTnI gold standard are notable. Because cMyC was not measured through the patients’ journey, it is a matter of speculation whether the outcome would have been different with cMyC as the adjudicating biomarker.

After iatrogenic or spontaneous AMI, cMyC appears more rapidly in the systemic circulation than either hs-cTnT or hs-cTnI.^16,20^ This finding is probably a result of a combination of cMyC’s greater myocardial abundance, distinct sarcomeric location, and loose association with myosin and actin.^16^ This biological distinctiveness of cMyC likely underpins the diagnostic advantage we observed over hs-cTnT/hs-cTnI in patients presenting <3 hours of symptom onset. Moreover, the more rapid appearance of cMyC in the systemic circulation after cardiac injury is also likely to explain the net reclassification improvement over both hs-cTnT and hs-cTnI.

No large prospective clinical trials compare the effect of different biomarkers of cardiac necrosis on clinical outcome. Nonetheless, it is interesting to speculate what effect the improved classification of events by cMyC could have in clinical practice. The current guidelines identify 3 risk groups, where only hs-cTn concentrations at the limit of detection or significantly above the 99th percentile clearly triage patients toward rule-out or rule-in of AMI, respectively.^6^ This outcome leaves a significant proportion of patients within the observe zone of clinical uncertainty requiring repeat cTn measurement and further investigation.^32^ In the current study, of the patients who ultimately did not have AMI, the proportion in the observe zone after the first measurement at presentation was 55.2% using hs-cTnT, 63.2% using hs-cTnI, and 46.1% using cMyC. It is expected that the greater diagnostic certainty afforded on a single-presentation blood draw by cMyC may reduce median time to discharge and costs of investigations.

As yet, near-patient, point-of-care devices have not been able to rule out AMI because they have struggled to achieve the required analytic sensitivity to measure low concentrations of cTnT or cTnI. In addition, the development of reliable large platform-based hs-cTn assays has proved more challenging than expected. Until now, only 2 manufacturers have overcome the difficulties of developing and introducing hs-cTn assays into clinical practice,^6^ of which 1 had major quality issues initially.^33–35^ These uncertainties and concerns have led to delays in the approval of these assays for clinical care in the United States.^36^ The US Food and Drug Administration has only recently ratified the use of the fifth-generation hs-cTnT assay.^37^ Because cMyC is more abundant and rises more rapidly, migration to a point-of-care format may be less challenging. Risk prediction appears grossly similar when comparing hs-cTn and cMyC and could therefore be performed on either. Notably, a cMyC level <10 ng/L (the threshold resembling 25 times the limit of detection) offers both high negative predictive value and 30-day mortality rates approaching 0.

Our study has a number of limitations. First, the diagnostic cutoffs for cMyC require external validation. Despite its size, a single cohort cannot entirely safeguard against calibration issues and is inherently subject to potential institutional bias. We have attempted to mitigate these risks by using both randomization and bootstrapping, but in an ideal scenario the findings require validation in an independent cohort. Second, the analyses within this manuscript are confined to the concentration of the necrosis biomarker on first blood draw. We have not analyzed the effect on the gray zone of repeat blood draws after set intervals. This area of active research has no consensus regarding resampling interval, magnitude of concentration change, use of absolute or relative change in concentration, or effect of assay vendor.^4,5,21,38–40^ Third, as a prospective diagnostic study, we cannot exactly quantify the clinical benefit associated with the use of cMyC as an alternative or addition to hs-cTn. Further cluster-randomized studies will be required to address this issue. Fourth, we cannot comment on the accuracy of cMyC among patients with terminal kidney failure on renal replacement therapy or ST elevation myocardial infarction because such patients were excluded from this study. Currently, biomarkers have no role in the assessment of patients with ST elevation myocardial infarction, and hence this group was not analyzed. Fifth, of 3029 patients recruited, 875 had no baseline cMyC measured. However, a comparison between the analyzed cohort and the excluded patient sample has not demonstrated substantial differences in baseline characteristics (Table III in the online-only Data Supplement). Sixth, in patients with low levels of cMyC (eg, the noncardiac chest pain group), we observed a significant difference in biomarker values dependent on certain underlying conditions (such as prior coronary artery disease) (Tables VII and VIII in the online-only Data Supplement). However, this effect is muted in patients with AMI and indeed did not negatively influence specificity. Finally, cMyC was measured using a research platform, whereas hs-cTnI and hs-cTnT were measured using widely available clinical laboratory analyzers. The sandwich immunoassay is comparable to the setup used to test for hs-cTn, but cMyC is not yet available on a random-access laboratory analyzer for routine clinical use.

In summary, cMyC is a promising new biomarker of myocardial necrosis, with overall discriminatory power comparable with the leading troponin assays in AMI diagnosis. A potential advantage of cMyC is its ability to more effectively rule out AMI at presentation, particularly among those presenting early after symptom onset.

## Sources of Funding

APACE was supported by research grants from the Swiss National Science Foundation, the European Union, the Swiss Heart Foundation, the Cardiovascular Research Foundation Basel, the University Hospital Basel, Abbott, Brahms, Biomerieux, Beckman Coulter, Nanosphere, Roche, Singulex, 8sense, and Siemens. The sponsors had no role in the design and conduct of the study; collection, management, analysis, and interpretation of the data; or preparation, review, and approval of the manuscript. This work was further supported by grants from the Medical Research Council (United Kingdom) (G1000737), Guy’s and St Thomas’ Charity (R060701, R100404), the British Heart Foundation (TG/15/1/31518, FS/15/13/31320), and the United Kingdom Department of Health through the National Institute for Health Research Biomedical Research Center award to Guy’s and St Thomas’ National Health Service Foundation Trust.

## Disclosures

Dr Twerenbold has received a research grant from the Swiss National Science Foundation (P300PB-167803) and speaker/consulting honoraria from Roche, Abbott, and BRAHMS. Dr Rubini has received speaker honoraria from Abbott. Dr Reichlin has received research grants from Goldschmidt-Jacobson-Foundation, the Swiss National Science Foundation (PASMP3-136995), the Swiss Heart Foundation, the University of Basel, the Professor Max Cloetta Foundation, the Uniscientia Foundation Vaduz, and the Department of Internal Medicine, University Hospital Basel; and speaker honoraria from BRAHMS and Roche. Dr Mueller has received research grants from the Swiss National Science Foundation, the Swiss Heart Foundation, the European Union, the Swiss Commission for Technology and Innovation, the Cardiovascular Research Foundation Basel, the University Hospital Basel, Abbott, Alere, Astra Zeneca, Beckman Coulter, BG Medicine, Biomerieux, BRAHMS, Critical Diagnostics, Nanosphere, Roche, Siemens, Singulex, Sphingotec, Department of Internal Medicine (University Hospital Basel), and 8sense; and speaker/consulting honoraria from Abbott, Alere, Astra Zeneca, Biomerieux, BMS, Boehringer Ingelheim, BRAHMS, Cardiorentis, Duke University, Eli Lilly, Novartis, Radiometer, Roche, Sanofi, Siemens, and Singulex. The other authors report no conflicts of interest. Millipore Sigma was contracted to undertake the analyses of cMyC on a fee-for-service basis and holds no commercial interest. Prof Marber is named as an inventor on a patent held by King’s College London for the detection of cMyC as a biomarker of myocardial injury.

## Supplementary Material

## References

[1] U.S. Department of Health and Human Services, Centers for Disease Control and Prevention, National Center for Health Statistics National Hospital Ambulatory Medical Care Survey: 2012 Emergency Department Summary Tables [published online ahead of print May 3, 2017].. http://www.cdc.gov/nchs/data/ahcd/nhamcs_emergency/2012_ed_web_tables.pdf.

[2] McManus DD, Gore J, Yarzebski J, Spencer F, Lessard D, Goldberg RJ (2011). Recent trends in the incidence, treatment, and outcomes of patients with STEMI and NSTEMI.. Am J Med.

[3] Katus HA, Remppis A, Neumann FJ, Scheffold T, Diederich KW, Vinar G, Noe A, Matern G, Kuebler W (1991). Diagnostic efficiency of troponin T measurements in acute myocardial infarction.. Circulation.

[4] Reichlin T, Hochholzer W, Bassetti S, Steuer S, Stelzig C, Hartwiger S, Biedert S, Schaub N, Buerge C, Potocki M, Noveanu M, Breidthardt T, Twerenbold R, Winkler K, Bingisser R, Mueller C (2009). Early diagnosis of myocardial infarction with sensitive cardiac troponin assays.. N Engl J Med.

[5] Amsterdam EA, Wenger NK, Brindis RG, Casey DE, Ganiats TG, Holmes DR, Jaffe AS, Jneid H, Kelly RF, Kontos MC, Levine GN, Liebson PR, Mukherjee D, Peterson ED, Sabatine MS, Smalling RW, Zieman SJ (2014). 2014 ACC/AHA guideline for the management of patients with non–ST-elevation acute coronary syndromes: a report of the American College of Cardiology/American Heart Association Task Force on Practice Guidelines.. Circulation.

[6] Roffi M, Patrono C, Collet JP, Mueller C, Valgimigli M, Andreotti F, Bax JJ, Borger MA, Brotons C, Chew DP, Gencer B, Hasenfuss G, Kjeldsen K, Lancellotti P, Landmesser U, Mehilli J, Mukherjee D, Storey RF, Windecker S, Baumgartner H, Gaemperli O, Achenbach S, Agewall S, Badimon L, Baigent C, Bueno H, Bugiardini R, Carerj S, Casselman F, Cuisset T, Erol Ç, Fitzsimons D, Halle M, Hamm C, Hildick-Smith D, Huber K, Iliodromitis E, James S, Lewis BS, Lip GY, Piepoli MF, Richter D, Rosemann T, Sechtem U, Steg PG, Vrints C, Luis Zamorano J, Management of Acute Coronary Syndromes in Patients Presenting without Persistent ST-Segment Elevation of the European Society of Cardiology (2016). 2015 ESC guidelines for the management of acute coronary syndromes in patients presenting without persistent ST-segment elevation: task force for the management of acute coronary syndromes in patients presenting without persistent ST-segment elevation of the European Society of Cardiology (ESC).. Eur Heart J.

[7] Reichlin T, Twerenbold R, Maushart C, Reiter M, Moehring B, Schaub N, Balmelli C, Rubini Gimenez M, Hoeller R, Sakarikos K, Drexler B, Haaf P, Osswald S, Mueller C (2013). Risk stratification in patients with unstable angina using absolute serial changes of 3 high-sensitive troponin assays.. Am Heart J.

[8] Boeddinghaus J, Reichlin T, Cullen L, Greenslade JH, Parsonage WA, Hammett C, Pickering JW, Hawkins T, Aldous S, Twerenbold R, Wildi K, Nestelberger T, Grimm K, Rubini-Gimenez M, Puelacher C, Kern V, Rentsch K, Than M, Mueller C (2016). Two-hour algorithm for triage toward rule-out and rule-in of acute myocardial infarction by use of high-sensitivity cardiac troponin I.. Clin Chem.

[9] Offer G, Moos C, Starr R (1973). A new protein of the thick filaments of vertebrate skeletal myofibrils: extractions, purification and characterization.. J Mol Biol.

[10] Fougerousse F, Delezoide AL, Fiszman MY, Schwartz K, Beckmann JS, Carrier L (1998). Cardiac myosin binding protein C gene is specifically expressed in heart during murine and human development.. Circ Res.

[11] The Human Protein Atlas MYBPC3 [published online ahead of print May 3, 2017].. http://www.proteinatlas.org/ENSG00000134571-MYBPC3/tissue.

[12] Kuster DWD, Barefield D, Govindan S, Sadayappan S (2013). A sensitive and specific quantitation method for determination of serum cardiac myosin binding protein-C by electrochemiluminescence immunoassay.. J Vis Exp.

[13] Kuster DW, Cardenas-Ospina A, Miller L, Liebetrau C, Troidl C, Nef HM, Möllmann H, Hamm CW, Pieper KS, Mahaffey KW, Kleiman NS, Stuyvers BD, Marian AJ, Sadayappan S (2014). Release kinetics of circulating cardiac myosin binding protein-C following cardiac injury.. Am J Physiol Heart Circ Physiol.

[14] Lynch TL, Sadayappan S (2014). Surviving the infarct: A profile of cardiac myosin binding protein-C pathogenicity, diagnostic utility, and proteomics in the ischemic myocardium.. Proteomics Clin Appl.

[15] Govindan S, Kuster DW, Lin B, Kahn DJ, Jeske WP, Walenga JM, Leya F, Hoppensteadt D, Fareed J, Sadayappan S (2013). Increase in cardiac myosin binding protein-C plasma levels is a sensitive and cardiac-specific biomarker of myocardial infarction.. Am J Cardiovasc Dis.

[16] Baker JO, Tyther R, Liebetrau C, Clark J, Howarth R, Patterson T, Möllmann H, Nef H, Sicard P, Kailey B, Devaraj R, Redwood SR, Kunst G, Weber E, Marber MS (2015). Cardiac myosin-binding protein C: a potential early biomarker of myocardial injury.. Basic Res Cardiol.

[17] Jacquet S, Yin X, Sicard P, Clark J, Kanaganayagam GS, Mayr M, Marber MS (2009). Identification of cardiac myosin-binding protein C as a candidate biomarker of myocardial infarction by proteomics analysis.. Mol Cell Proteomics.

[18] Marjot J, Kaier TE, Martin ED, Reji SS, Copeland O, Iqbal M, Goodson B, Hamren S, Harding SE, Marber MS (2017). Quantifying the release of biomarkers of myocardial necrosis from cardiac myocytes and intact myocardium.. Clin Chem.

[19] Marjot J, Liebetrau C, Goodson RJ, Kaier T, Weber E, Heseltine P, Marber MS (2016). The development and application of a high-sensitivity immunoassay for cardiac myosin-binding protein C.. Transl Res.

[20] Kaier TE, Anand A, Shah AS, Mills NL, Marber M (2016). Temporal relationship between cardiac myosin-binding protein C and cardiac troponin I in type 1 myocardial infarction.. Clin Chem.

[21] Rubini Gimenez M, Twerenbold R, Reichlin T, Wildi K, Haaf P, Schaefer M, Zellweger C, Moehring B, Stallone F, Sou SM, Mueller M, Denhaerynck K, Mosimann T, Reiter M, Meller B, Freese M, Stelzig C, Klimmeck I, Voegele J, Hartmann B, Rentsch K, Osswald S, Mueller C (2014). Direct comparison of high-sensitivity-cardiac troponin I vs. T for the early diagnosis of acute myocardial infarction.. Eur Heart J.

[22] Reichlin T, Hochholzer W, Stelzig C, Laule K, Freidank H, Morgenthaler NG, Bergmann A, Potocki M, Noveanu M, Breidthardt T, Christ A, Boldanova T, Merki R, Schaub N, Bingisser R, Christ M, Mueller C (2009). Incremental value of copeptin for rapid rule out of acute myocardial infarction.. J Am Coll Cardiol.

[23] Haaf P, Reichlin T, Twerenbold R, Hoeller R, Rubini Gimenez M, Zellweger C, Moehring B, Fischer C, Meller B, Wildi K, Freese M, Stelzig C, Mosimann T, Reiter M, Mueller M, Hochgruber T, Sou SM, Murray K, Minners J, Freidank H, Osswald S, Mueller C (2014). Risk stratification in patients with acute chest pain using three high-sensitivity cardiac troponin assays.. Eur Heart J.

[24] Thygesen K, Alpert JS, White HD, Joint ESC/ACCF/AHA/WHF Task Force for the Redefinition of Myocardial Infarction (2007). Universal definition of myocardial infarction.. Circulation.

[25] Thygesen K, Mair J, Giannitsis E, Mueller C, Lindahl B, Blankenberg S, Huber K, Plebani M, Biasucci LM, Tubaro M, Collinson P, Venge P, Hasin Y, Galvani M, Koenig W, Hamm C, Alpert JS, Katus H, Jaffe AS, Study Group on Biomarkers in Cardiology of ESC Working Group on Acute Cardiac Care (2012). How to use high-sensitivity cardiac troponins in acute cardiac care.. Eur Heart J.

[26] Apple FS, Wu AH, Jaffe AS (2002). European Society of Cardiology and American College of Cardiology guidelines for redefinition of myocardial infarction: how to use existing assays clinically and for clinical trials.. Am Heart J.

[27] Apple FS, Jesse RL, Newby LK, Wu AH, Christenson RH, Cannon CP, Francis G, Morrow DA, Ravkilde J, Storrow AB, Tang W, Jaffe AS, Mair J, Ordonez-Llanos J, Pagani F, Panteghini M, Tate J, IFCC Comittee on Standardization of Markers of Cardiac Damege; National Academy of Clinical Biochemistry (2007). National Academy of Clinical Biochemistry and IFCC Committee for Standardization of Markers of Cardiac Damage Laboratory Medicine Practice Guidelines: analytical issues for biochemical markers of acute coronary syndromes.. Clin Chem.

[28] Pickering JW, Endre ZH (2012). New metrics for assessing diagnostic potential of candidate biomarkers.. Clin J Am Soc Nephrol.

[29] Harrell FE, Califf RM, Pryor DB, Lee KL, Rosati RA (1982). Evaluating the yield of medical tests.. JAMA.

[30] Pencina MJ, D’Agostino RB, D’Agostino RB, Vasan RS (2008). Evaluating the added predictive ability of a new marker: from area under the ROC curve to reclassification and beyond.. Stat Med.

[31] Thygesen K, Alpert JS, Jaffe AS, Simoons ML, Chaitman BR, White HD, the Writing Group on behalf of the Joint ESC/ACCF/AHA/WHF Task Force for the Universal Definition of Myocardial Infarction (2012). Third universal definition of myocardial infarction.. Circulation.

[32] Nestelberger T, Wildi K, Boeddinghaus J, Twerenbold R, Reichlin T, Giménez MR, Puelacher C, Jaeger C, Grimm K, Sabti Z, Hillinger P, Kozhuharov N, du Fay de Lavallaz J, Pinck F, Lopez B, Salgado E, Miró Ò, Bingisser R, Lohrmann J, Osswald S, Mueller C (2016). Characterization of the observe zone of the ESC 2015 high-sensitivity cardiac troponin 0h/1h-algorithm for the early diagnosis of acute myocardial infarction.. Int J Cardiol.

[33] Hallermayer K, Jarausch J, Menassanch-Volker S, Zaugg C, Ziegler A (2013). Implications of adjustment of high-sensitivity cardiac troponin T assay.. Clin Chem.

[34] Kavsak PA, Hill SA, McQueen MJ, Devereaux PJ (2013). Implications of adjustment of high-sensitivity cardiac troponin T assay.. Clin Chem.

[35] Wildi K, Twerenbold R, Jaeger C, Rubini Giménez M, Reichlin T, Stoll M, Hillinger P, Puelacher C, Boeddinghaus J, Nestelberger T, Grimm K, Grob M, Rentsch K, Arnold C, Mueller C (2016). Clinical impact of the 2010-2012 low-end shift of high-sensitivity cardiac troponin T.. Eur Heart J Acute Cardiovasc Care.

[36] Apple FS, Hollander J, Wu AH, Jaffe AS (2014). Improving the 510(k) FDA process for cardiac troponin assays: in search of common ground.. Clin Chem.

[37] U.S. Food and Drug Administration 510(k) Premarket Notification [published online ahead of print April 3, 2017].. https://www.accessdata.fda.gov/scripts/cdrh/cfdocs/cfPMN/pmn.cfm?ID=K162895.

[38] Haaf P, Drexler B, Reichlin T, Twerenbold R, Reiter M, Meissner J, Schaub N, Stelzig C, Freese M, Heinzelmann A, Meune C, Balmelli C, Freidank H, Winkler K, Denhaerynck K, Hochholzer W, Osswald S, Mueller C (2012). High-sensitivity cardiac troponin in the distinction of acute myocardial infarction from acute cardiac noncoronary artery disease.. Circulation.

[39] Hoeller R, Rubini Giménez M, Reichlin T, Twerenbold R, Zellweger C, Moehring B, Wildi K, Freese M, Stelzig C, Hartmann B, Stoll M, Mosimann T, Reiter M, Haaf P, Mueller M, Meller B, Hochgruber T, Balmelli C, Sou SM, Murray K, Freidank H, Steuer S, Minners J, Osswald S, Mueller C (2013). Normal presenting levels of high-sensitivity troponin and myocardial infarction.. Heart.

[40] Keller T, Zeller T, Ojeda F, Tzikas S, Lillpopp L, Sinning C, Wild P, Genth-Zotz S, Warnholtz A, Giannitsis E, Möckel M, Bickel C, Peetz D, Lackner K, Baldus S, Münzel T, Blankenberg S (2011). Serial changes in highly sensitive troponin I assay and early diagnosis of myocardial infarction.. JAMA.

