# Supplementary material for: Direct Comparison of Cardiac Myosin-Binding Protein C With Cardiac Troponins for the Early Diagnosis of Acute Myocardial Infarction
Source: Circulation. 2017 Oct 16;136(16):1495–508. doi: 10.1161/CIRCULATIONAHA.117.028084 (PMC5642333; doi:10.1161/CIRCULATIONAHA.117.028084)
Supplement: Supplementary file 1 [file cir-136-1495-s001.pdf]

Supplemental Material: Direct comparison of cardiac myosin-binding protein C with cardiac troponins for the early diagnosis of acute myocardial infarction

Running title: Cardiac Myosin-binding protein C in the diagnosis of AMI

Thomas E Kaier, MD, MBA[1*]; Raphael Twerenbold, MD[2,3*]; Christian Puelacher, MD[2]; Jack Marjot, MBBS BSc[1]; Nazia Imambaccus[1]; Jasper Boeddinghaus, MD[2]; Thomas Nestelberger, MD[2]; Patrick Badertscher, MD[2]; Zaid Sabti, MD[2]; Maria Rubini Giménez, MD[2,3]; Karin Wildi, MD[2,4]; Petra Hillinger, MD[2]; Karin Grimm, MD[2]; Sarah Loeffel[2]; Samyut Shrestha, MD[2]; Dayana Flores Widmer, MD[2]; Janosch Cupa, MD[2]; Nikola Kozhuharov, MD[2]; Òscar Miró, MD[5,6]; F. Javier Martín-Sánchez, MD[6,7]; Beata Morawiec MD[6,8]; Katharina Rentsch, PhD[9]; Jens Lohrmann, MD[2]; Wanda Kloos, MD[2]; Stefan Osswald, MD[2]; Tobias Reichlin, MD[2]; Ekkehard Weber, PhD[10]; Michael Marber, MD, PhD[1#]; Christian Mueller, MD[2,6#]

[1]King's College London BHF Centre, The Rayne Institute, St Thomas' Hospital, London, UK
[2]Department of Cardiology and Cardiovascular Research Institute Basel (CRIB), University Hospital Basel, Switzerland
[3]Department of General and Interventional Cardiology, University Heart Center Hamburg, Hamburg, Germany
[4]Emergency department, CIBERES ISC III, Hospital del Mar – IMIM, Barcelona, Spain
[5]Emergency department, Hospital Clinic, Barcelona, Spain
[6]Global Research in Acute Conditions (GREAT) network
[7]Emergency department, Hospital Clinico San Carlos, Madrid, Spain
[8]2nd Cardiology department, Zabrze, University Silesia, Katowice, Poland
[9]Laboratory Medicine, University Hospital Basel, Switzerland
[10]Institute of Physiological Chemistry, Martin Luther University Halle-Wittenberg, Halle, Germany

*Both authors have contributed equally and should be considered first author

[#]Both research groups have contributed equally

Corresponding author: Professor Michael Marber, The Rayne Institute, 4th Floor Lambeth Wing, St Thomas' Hospital, Westminster Bridge Road, London SE1 7EH, UK; Tel: +44-(0)20-7188 1008, Fax: +44-(0)20-7188 0970. email: mike.marber@kcl.ac.uk

Supplemental Methods

Routine clinical assessment

All patients underwent a clinical assessment that included medical history, physical examination, 12-lead ECG, pulse oximetry, standard blood test, and chest radiography according to local protocols and in accordance with the guidelines of the European Society of Cardiology (ESC).[1] Levels of cTn were measured at presentation and serially thereafter as long as clinically indicated. Treatment of patients was left to discretion of the attending physician.

Adjudication of the final diagnosis

AMI was defined and cTn levels interpreted as recommended in current guidelines.[2-5] In brief, AMI was diagnosed when there was evidence of myocardial necrosis with a significant rise and/or fall in a clinical setting consistent with myocardial ischemia. Patients with AMI were further subdivided into type 1 AMI (primary coronary events) and type 2 AMI (ischemia due to increased demand or decreased supply, for example tachyarrhythmia or hypertensive crisis).[2,6]

The adjudication of final diagnoses was performed centrally in the core lab (University Hospital Basel) for all patients incorporating levels of hs-cTnT (see test characteristics above). More specifically, two independent cardiologists not directly involved in patient care reviewed all available medical records (including patient history, physical examination, results of laboratory testing including hs-cTnT levels, radiologic testing, ECG, echocardiography, cardiac exercise test, lesion severity and morphology in coronary angiography, discharge summary) pertaining to the patient from the time of ED presentation to 90-day follow-up. Late samples were available for adjudication of final diagnosis in all patients. In general, serial sampling was performed until at least 6h after presentation to the ED or onset of chest.[6] In situations of diagnostic disagreement, cases were reviewed and adjudicated in conjunction with a third cardiologist. While discharge diagnoses often were correct and in agreement with the final adjudicated diagnosis, there were also cases where those diagnoses needed to be revised, most often because more information became available from medical testing during early follow-up, and more rarely, because the discharge diagnosis was not in agreement with the Universal Definition of AMI.

The 99th percentile (14ng/L) was used as cut-off for myocardial necrosis. Absolute cTn changes were used to determine significant changes based on the diagnostic superiority of absolute over relative changes.[7-12] Based on studies of the biological variation of cTn {Wu:2008bf, Vasile:2010fc} as well as on data from previous chest pain cohort studies {Reichlin:2011iu, Hammarsten:2012cv}, a significant absolute change was defined as a rise or fall of at least 10ng/L within six hours, or, in an assumption of linearity, as an absolute change of 6ng/L within three hours. Predefined alternative diagnoses included "unstable angina" (UA), "Cardiac symptoms of origin other than coronary artery disease" and "non-cardiac chest pain".

Clinical Care: The (hs)-cTn assays and cut-off levels used for local clinical care

Routine clinical care comprised five different cTn assays at the different hospitals and at the different recruitment periods. The cTn assays used clinically in most of the participating institutions changed during the study from a conventional cTn assay to the hs-cTnT assay. In order to take advantage of the higher sensitivity and higher overall diagnostic accuracy offered by the hs-cTnT assay, patients were adjudicated using the hs-cTnT values in all patients. In patients in whom clinically a conventional cTn assay was used, the conventional cTn values and the hs-cTnT values were available for the adjudication. In patients in whom clinically the hs-cTnT assay was used, only the hs-cTnT values were available for the adjudication.

The following conventional cTn assays were used: For the Roche cTnT 4th generation assay, the 10% CV level is 0.035ug/l. The laboratories of the participating sites reported only two decimals; therefore 0.04ug/l was used as a cut-off for myocardial necrosis. In order to fulfil the criteria of a significant change (30% of 99th percentile or 10% CV level), a patient would e.g. need to have a level of $<$0.01ug/l at presentation and 0.04ug/l at 6h. A patient would also qualify if the first level is 0.02ug/l and the second 0.04ug/l. A patient would not fulfil the criteria if the first level is 0.03ug/l and the second is 0.04ug/l. If the first level is 0.04ug/l, the second level needs to be at least 0.06ug/l.

For the Abbott Axsym cTnI ADV, the 10% CV level is 0.16ug/l. A patient having 0.16ug/l at presentation would meet the criteria for significant change if the second was $\geq$0.21ug/l. A patient having $<$0.12ug/l at presentation (limit of detection) would qualify if the second is $>$0.16ug/l.

For the Beckmann Coulter Accu cTnI, the 10% CV level is 0.06ug/l. A patient having 0.06ug/l at presentation would qualify if the second is $\geq$0.08ug/l. A patient having 0.05 at presentation would qualify if the second is 0.07ug/l, but not 0.06ug/l. A patient having undetectable cTnI (cTnI$<$0.01ug/l) at presentation would qualify if the second is $\geq$0.06ug/l.

For the Siemens Dimension Vista s-cTnI, the 10% CV level is 40ng/L. The limit of detection is 15ng/L and the 99th percentile is 45ng/L. An absolute change of 20ng/L or more within 3-6h was considered significant.

For Elecsys hs-cTnT measured clinically, the same change criteria were applied as for hs-cTnT measured from the study blood samples.

Central adjudication: Definition of rise and/or fall in high-sensitivity cardiac troponin T (hs-cTnT)

Absolute changes in hs-cTnT were used to determine significant changes based on the diagnostic superiority of absolute over relative changes.[7-12] Based on studies of the biological variation of cTn [13,14] as well as on data from previous chest

pain cohort studies [15,16], a significant absolute change was defined as a rise or fall of at least 10 ng/L within 6 hours or an absolute change of 6 ng/L within 3 hours. If later clinical samples (e.g., at 24, 48, or 72 hours) revealed a lower hs-cTnT level than that measured during the period of sampling in the ED, the later level was considered the true baseline level for the calculation of the change criteria.

Measurement of high-sensitivity cardiac troponin I, high-sensitivity cardiac troponin T and sensitive cardiac troponin I

After collection and subsequent centrifugation, samples were frozen at -80ºC until assayed in a blinded fashion in a dedicated core laboratory. The Roche hs-cTnT assay was measured on the Elecsys 2010 (Roche Diagnostics). The limit of blank and LoD were determined to be 3 and 5 ng/L, respectively. The 99th-percentile of a healthy reference population was reported at 14 ng/L with an imprecision corresponding to 10% CV at 13 ng/L.[17] This study does not include any measurements with hs-cTnT lots that required the revision of the calibration curve.[18-22] The Abbott hs-cTnI assay used was the final pre-commercial release version of the ARCHITECT High Sensitive STAT Troponin I assay (Abbott Laboratories, Abbott Park, IL, USA). Samples were thawed, mixed, and centrifuged (for 30 min at 3000 RCF and 4ºC for serum samples or for 10 min, twice, at 3000 RCF for plasma samples) prior to analysis and according to manufacturer's instructions. The hs-cTnI assay has a 99th percentile concentration of 26·2 ng/L with a corresponding coefficient of variation (CV) of <5% and a limit of detection (LoD) of 1.9 ng/L.[23] The cTnI-ultra assay was performed with the use of the ADVIA Centaur immunoassay system (Siemens). Limit of detection is 6 ng/L; a 10% coefficient of variation was reported at 30 ng/L with the 99th percentile cut-off point of 40 ng/L.[24,25] Calculation of the glomerular filtration rate was performed using the abbreviated Modification of Diet in Renal disease formula.[26]

Measurement of cardiac myosin-binding protein C

We have previously described the creation, biophysical selection and organ specificity of mouse monoclonal antibodies recognising cardiac-restricted epitopes within the N-terminus of cMyC.[27] Two of these antibodies, 1A4 and 3H8, were used to create a sensitive sandwich immunoassay. In brief, Magnetic microparticles (MPs) for capture were prepared by binding 25µg of mouse monoclonal (1A4) per mg of MPs. The coated MPs were diluted in assay buffer (proprietary mix with custom 450mM NaCl and 0.5% Triton X-100) to 100µg/mL. Due to sample volume constraints, serum, plasma or analyte (recombinant C0C2 domain of cMyC)[27] were diluted 2.2 fold with standard diluent and 100µL added per well of a 96-well assay plate. Samples or standards were then exposed to 100µL of coated MPs and agitated for 2 hours at 25°C. MPs were retained via a magnetic bed with unbound material removed in a single wash step. Fluorescently-labelled mouse monoclonal (3H8) detection antibody was diluted in assay buffer to 100ng/mL. To each well, 20µL of detection antibody was added and the MPs agitated for 1 hour at 25°C, retained via a magnetic bed and then washed 4 times to

remove any unbound detection reagent. The MPs were then transferred to a new plate and all buffer was aspirated. The MPs were then exposed to 20µL/well of elution buffer B for 5 minutes at 25°C before transfer to a 384-well plate containing 10µL/well of neutralization buffer D. Fluorescent label was then detected by single molecule counting using the Erenna system with a dwell time of 60s per well. Three signal outputs were obtained from the Erenna System: Detected Events (DEs; low end signal), Event Photons (EPs; low end and higher end signal), and Total Photons (TPs; high end signal).

Supplemental Tables

Table S1. Comparison of biomarkers in patients excluded because of uncertain final diagnosis (e.g. patients discharged based on negative result on conventional cTn assay, who then tested positive on high-sensitivity cTn assay); comparison is performed for all patients with a measured cMyC at baseline (N=60) and all patients including missing values (N=92)

Biomarker	N	Median ng/L [IQR]
**cMyC at 0h**	60	36 [24-62]
**hs-cTnI at 0h**	56	11 [7-21]
**hs-cTnT at 0h**	60	21 [16-28]
**All patients**	92	
**cMyC at 0h**	60	36 [24-62]
**hs-cTnI at 0h**	78	11 [6-19]
**hs-cTnT at 0h**	92	22 [17-29]

Table S2. Demographics: group qualifying for primary analysis (n=1954) vs patients excluded due to missing cMyC values at baseline

Demographics	All patients (n = 1954)	Excluded patients (n=875)	p value* for comparison
Age, years	62 ± 16	59 ± 16	<0.001
Male	1341 (69)	587 (67)	0.441
**Risk factors**			
Hypertension	1247 (64)	384 (44)	<0.001
Hyperlipidaemia	992 (51)	421 (48)	0.206
Diabetes mellitus	348 (18)	136 (16)	0.155
Current smoking	476 (24)	244 (28)	0.051
History of smoking	1194 (61)	553 (63)	0.297
**History**			
Coronary artery disease	710 (36)	272 (31)	0.008
Previous myocardial infarction	474 (24)	199 (23)	0.408
Previous revascularisation (CABG or PCI)	553 (28)	237 (27)	0.535
Peripheral artery disease	119 (6)	52 (6)	0.947
Previous stroke	100 (5)	53 (6)	0.352
**Vital status**			
Heart rate, beats/min	79 ± 20	81 ± 21	0.234
Systolic blood pressure, mm Hg	144 ± 24	143 ± 25	0.711
Diastolic blood pressure, mm Hg	82 ± 15	82 ± 15	0.569
**Electrocardiographic findings**			
ST-segment depression	193 (10)	75 (9)	0.313
T-wave inversion	260 (13)	89 (10)	0.026
No significant electrocardiographic abnormalities	1469 (75)	681 (79)	0.193
**Laboratory assessment**			
Estimated glomerular filtration rate, ml/min/1.73m2†	84 ± 26	87 ± 25	0.008
**Presentation time**			
Time since chest pain onset, hours	5 [3, 12]	4 [1, 9]	<0.001
Time since chest pain peak, hours	3 [2, 7]	2 [5, 5]	<0.001

Legend: * p values for comparison included versus excluded patient groups; data are expressed as medians [1st quartile, 3rd quartile] or means ± standard deviation, for categorical variables as numbers (percentages); CABG = Coronary Artery Bypass Graft; PCI = Percutaneous Coronary Intervention; † glomerular filtration rate was estimated using the Modification of Diet in Renal Disease (MDRD) formula

Table S3. STARD checklist for studies of diagnostic accuracy

Section & Topic	No	Item	Reported on page #
TITLE OR ABSTRACT	1	Identification as a study of diagnostic accuracy using at least one measure of accuracy (such as sensitivity, specificity, predictive values, or AUC)	1, 3
ABSTRACT	2	Structured summary of study design, methods, results, and conclusions (for specific guidance, see STARD for Abstracts)	3-4
INTRODUCTION	3	Scientific and clinical background, including the intended use and clinical role of the index test	5-6
	4	Study objectives and hypotheses	5-6
METHODS			
Study design	5	Whether data collection was planned before the index test and reference standard were performed (prospective study) or after (retrospective study)	7
Participants	6	Eligibility criteria	7
	7	On what basis potentially eligible participants were identified (such as symptoms, results from previous tests, inclusion in registry)	7
	8	Where and when potentially eligible participants were identified (setting, location and dates)	7-8
	9	Whether participants formed a consecutive, random or convenience series	7
Test methods	10a	Index test, in sufficient detail to allow replication	8-9 and supplement
	10b	Reference standard, in sufficient detail to allow replication	8-9 and supplement
	11	Rationale for choosing the reference standard (if alternatives exist)	n/a
	12a	Definition of and rationale for test positivity cut-offs or result categories of the index test, distinguishing pre-specified from exploratory	9-10
	12b	Definition of and rationale for test positivity cut-offs or result categories of the reference standard, distinguishing pre-specified from exploratory	9-10
	13a	Whether clinical information and reference standard results were available to the performers/readers of the index test	7-9
	13b	Whether clinical information and index test results were available to the assessors of the reference standard	7-9
Analysis	14	Methods for estimating or comparing measures of diagnostic accuracy	8-10
	15	How indeterminate index test or reference standard results were handled	8-10
	16	How missing data on the index test and reference standard were handled	9
	17	Any analyses of variability in diagnostic accuracy, distinguishing pre-specified from exploratory	9
	18	Intended sample size and how it was determined	n/a
RESULTS			
Participants	19	Flow of participants, using a diagram	7 and Figures S1 + 2S
	20	Baseline demographic and clinical characteristics of participants	13, table 1

	21a	Distribution of severity of disease in those with the target condition	13, figure 2, suppl table 3S
	21b	Distribution of alternative diagnoses in those without the target condition	13, table 1
	22	Time interval and any clinical interventions between index test and reference standard	n/a
Test results	23	Cross tabulation of the index test results (or their distribution) by the results of the reference standard	13-16, tables 2+3, figures 3A+3B, suppl tables 4S+5S
	24	Estimates of diagnostic accuracy and their precision (such as 95% confidence intervals)	14
	25	Any adverse events from performing the index test or the reference standard	14-15
DISCUSSION			
	26	Study limitations, including sources of potential bias, statistical uncertainty, and generalisability	20
	27	Implications for practice, including the intended use and clinical role of the index test	2, 18-21
OTHER INFORMATION			
	28	Registration number and name of registry	4, 7
	29	Where the full study protocol can be accessed	7
	30	Sources of funding and other support; role of funders	22-24

Table S4. Demographics for derivation and validation cohorts

Demographics	All patients (n = 1954)	Derivation (n = 586)	Validation (n = 1368)	p value* for comparison
Age, years	62 ± 16	62 ± 16	62 ± 16	0.777
Male	1341 (69)	393 (67)	948 (69)	0.357
Acute Myocardial Infarction	340 (17)	107 (18)	233 (17)	0.512
**Risk factors**				
Hypertension	1247 (64)	362 (62)	885 (65)	0.239
Hyperlipidaemia	992 (51)	290 (49)	702 (51)	0.489
Diabetes mellitus	348 (18)	99 (17)	249 (18)	0.505
Current smoking	476 (24)	148 (25)	328 (24)	0.602
History of smoking	1194 (61)	372 (63)	864 (63)	0.906
**History**				
Coronary artery disease	710 (36)	200 (34)	510 (37)	0.202
Previous myocardial infarction	474 (24)	136 (23)	338 (25)	0.515
Previous revascularisation (CABG or PCI)	553 (28)	153 (26)	400 (29)	0.176
Peripheral artery disease	119 (6)	33 (6)	86 (6)	0.652
Previous stroke	100 (5)	27 (5)	73 (5)	0.577
**Vital status**				
Heart rate, beats/min	79 ± 20	80 (20)	79 (21)	0.895
Systolic blood pressure, mm Hg	144 ± 24	145 ± 25	143 ± 24	0.058
Diastolic blood pressure, mm Hg	82 ± 15	82 ± 15	82 ± 15	0.765
**Electrocardiographic findings**				
ST-segment depression	193 (10)	53 (9)	140 (10)	0.475
T-wave inversion	260 (13)	64 (11)	196 (14)	0.05
No significant electrocardiographic abnormalities	1469 (75)	456 (80)	1013 (76)	0.075
**Laboratory assessment**				
Estimated glomerular filtration rate, ml/min/1.73m2†	84 ± 26	84 ± 25	84 ± 26	0.441
**Presentation time**				
Time since chest pain onset, hours	5 [3, 12]	5 [2, 12]	5 [3, 12]	0.804
Time since chest pain peak, hours	3 [2, 7]	4 [2, 7]	3 [2, 7]	0.528

Legend: * p values for comparison validation to derivation cohort; data are expressed as medians [1st quartile, 3rd quartile] or means ± standard deviation, for categorical variables as numbers (percentages); CABG = Coronary Artery Bypass Graft; PCI = Percutaneous Coronary Intervention; † glomerular filtration rate was estimated using the Modification of Diet in Renal Disease (MDRD) formula

Table S5. Blood concentrations of cMyC, hs-cTnT, hs-cTnI and s-cTnI at presentation in the five diagnostic categories

Adjudicated diagnosis	cMyC (ng/L)	hs-cTnT (ng/L)	hs-cTnI (ng/L)	s-cTnI (mg/L)
**AMI**	237 [71-876]	62 [28-139]	97 [21-456]	0.175 [0.039-0.722]
**Unstable angina**	21 [13-43]	11 [7-17]	6 [4-12]	0.009 [0.005-0.020]
**cardiac symptoms of origin other than coronary artery disease**	33 [12-96]	15 [7-32]	10 [4-30]	0.017 [0.005-0.044]
**non-cardiac symptoms**	10 [6-19]	6 [4-10]	3 [2-5]	0.005 [0.004-0.011]
**symptoms of unknown origin**	11 [7-16]	6 [3-10]	3 [2-5]	0.005 [0.001-0.010]

Legend: AMI = acute myocardial infarction; data is quoted in median ng/L [Interquartile Range] for cMyC and hs-cTn assays, and mg/L [IQR] for s-cTnI

Table S6. Blood concentrations of biomarkers above 99th centiles at presentation

cMyC >87 ng/L	AMI	UA	non-coronary	non-cardiac	unknown	p	N
	N=237	N=18	N=72	N=22	N=1		
**cMyC at 0h**	559 [215-1228]	135 [113-207]	168 [120-329]	157 [101-222]	96 [96-96]	<0.001	350
**hs-cTnI at 0h**	230 [74-725]	42 [17-74]	81 [34-227]	29 [15-43]	3 [3-3]	<0.001	327
**hs-cTnT at 0h**	92 [51-182]	29 [24-43]	50 [35-88]	26 [19-47]	8 [8-8]	<0.001	337
**adjusted R^2: cMyC and hs-cTnI 0.230, cMyC and hs-cTnT 0.504, hs-cTnT and hs-cTnI 0.608**							

hs-cTnI >26 ng/L	AMI	UA	non-coronary	non-cardiac	unknown	p	N
	N=226	N=21	N=67	N=30	N=0		
**cMyC ng/L at 0h**	524 [208-1230]	60 [30-134]	158 [99-344]	75 [36-167]	NA	<0.001	344
**hs-cTnI ng/L at 0h**	235 [84-739]	60 [53-98]	91 [49-229]	42 [30-65]	NA	<0.001	344
**hs-cTnT ng/L at 0h**	93 [54-183]	22 [14-32]	50 [35-88]	27 [15-47]	NA	<0.001	332
**adjusted R^2: cMyC and hs-cTnI 0.230, cMyC and hs-cTnT 0.528, hs-cTnT and hs-cTnI 0.602**							

hs-cTnT >14 ng/L	AMI	UA	non-coronary	non-cardiac	unknown	p	N
	N=290	N=63	N=135	N=150	N=0		
**cMyC ng/L at 0h**	328 [97-998]	48 [24-83]	91 [46-165]	34 [19-57]	NA	<0.001	638
**hs-cTnI ng/L at 0h**	134 [33-557]	14 [7-38]	28 [14-87]	10 [6-19]	NA	<0.001	600
**hs-cTnT ng/L at 0h**	70 [36-147]	21 [17-26]	31 [20-52]	20 [16-27]	NA	<0.001	638
**adjusted R^2: cMyC and hs-cTnI 0.274, cMyC and hs-cTnT 0.567, hs-cTnT and hs-cTnI 0.617**							

Legend: AMI = acute myocardial infarction; UA = unstable angina; data is quoted in median ng/L [Interquartile Range]

Table S7. Non-Cardiac sources of cMyC variation

	AMI group (N=340)			p	N
**Gender - male vs female**	207 [62-814]	vs	361 [91-1006]	0.096	256
**Age - <65 vs ≥65**	237 [62-938]	vs	237 [74-826]	0.925	122
**Body Mass Index (BMI) - <30 vs ≥30**	264 [72-898]	vs	219 [76-616]	0.414	257
**Hypertension - absent vs present**	272 [64-885]	vs	230 [73-874]	0.935	71
**Hyperlipidaemia - absent vs present**	321 [92-840]	vs	211 [57-887]	0.140	113
**Diabetes mellitus - absent vs present**	282 [75-1004]	vs	182 [64-535]	0.059	245
**Current smoking - absent vs present**	257 [66-894]	vs	213 [78-822]	0.681	249
**History of smoking - absent vs present**	219 [70-837]	vs	274 [72-894]	0.701	198
**Coronary artery disease - absent vs present**	308 [79-973]	vs	206 [60-785]	0.135	166
**Estimated glomerular filtration rate, ml/min/1.73m2* - <60 vs ≥60**	345 [87-953]	vs	208 [60-828]	0.111	101
	**Non-cardiac chest pain group (N=1052)**				
**Gender – male vs female**	10 [6-19]	vs	10 [5-18]	0.108	716
**Age - <65 vs ≥65**	8 [5-13]	vs	18 [11-32]	<0.001	701
**Body Mass Index (BMI) - <30 vs ≥30**	10 [6-19]	vs	11 [6-21]	0.282	815
**Hypertension – absent vs present**	7 [5-12]	vs	14 [9-29]	<0.001	509
**Hyperlipidaemia – absent vs present**	8 [5-15]	vs	14 [8-28]	<0.001	634
**Diabetes mellitus – absent vs present**	10 [6-17]	vs	16 [10-35]	<0.001	916
**Current smoking – absent vs present**	11 [7-21]	vs	8 [5-14]	<0.001	769
**History of smoking – absent vs present**	10 [6-17]	vs	13 [7-23]	<0.001	707
**Coronary artery disease – absent vs present**	9 [6-15]	vs	18 [11-32]	<0.001	784
**Estimated glomerular filtration rate, ml/min/1.73m2* - <60 vs ≥60**	30 [16-53]	vs	9 [6-16]	<0.001	107

Legend: MI = myocardial infarction, based on the adjudicated gold-standard diagnosis; CABG = Coronary Artery Bypass Graft; PCI = Percutaneous Coronary Intervention; data is quoted in median [Interquartile Range]; N = number of patients with the condition on the left-hand side of the demographic factors (e.g. 'Hypertension – absent in 509 patients'); *glomerular filtration rate was estimated using the Modification of Diet in Renal Disease (MDRD) formula

Table S8. Multiple regression to determine influence of baseline variables on cMyC levels

**Non-cardiac chest pain group**	R^2	**B**	**SE B**	β_i	**p**
	0.077				
**Constant**		-32.439	11.893		0.006
**Hypertension**		2.040	3.860	0.020	0.597
**Hyperlipidaemia**		1.326	4.127	0.013	0.748
**Diabetes mellitus**		1.139	5.047	0.007	0.822
**Current smoking**		-1.914	3.978	-0.017	0.631
**History of smoking**		-3.531	3.680	-0.033	0.338
**Coronary artery disease**		9.658	4.552	0.083	0.034
**Creatinine on admission**		0.357	0.072	0.157	0.000
**Age**		0.399	0.113	0.127	0.000
**Body Mass Index (BMI)**		-0.007	0.328	-0.001	0.982
	0.075				
**Constant**		-34.087	7.050		0.000
**Coronary artery disease**		10.680	3.662	0.093	0.004
**Creatinine on admission**		0.351	0.070	0.155	0.000
**Age**		0.428	0.099	0.137	0.000

**AMI group**	R^2	**B**	**SE B**	β_i	**p**
	0.028				
**Constant**		516.589	496.148		0.299
**Hypertension**		-1.724	121.878	-0.001	0.989
**Hyperlipidaemia**		141.837	106.276	0.082	0.183
**Diabetes mellitus**		-236.237	108.644	-0.129	0.030
**Current smoking**		-30.010	136.079	-0.016	0.826
**History of smoking**		15.745	107.937	0.010	0.884
**Coronary artery disease**		-175.913	102.811	-0.108	0.088
**Creatinine on admission**		0.727	0.933	0.045	0.436
**Age**		-0.459	4.339	-0.007	0.916
**Body Mass Index (BMI)**		4.402	11.964	0.023	0.713
	0.014				
**Constant**		674.182	52.552		0.000
**Diabetes mellitus**		-220.143	100.580	-0.119	0.029

Legend: AMI = Acute Myocardial Infarction; R^2 = fit of the regression model; B = beta estimate; SE B = standard errors of beta estimate; β_i = standardized beta estimate

Table S9. Derivation cohort – hs-cTnT

**Initial model**	New model - cMyC (10/120) - Derivation cohort					
**hs-cTnT**	No AMI (n=465)			AMI (n=103)		
	Rule-out	Observe	Rule-in	Rule-out	Observe	Rule-in
**Rule-out**	105	28	0	0	0	0
**Observe**	95	221	8	0	41	10
**Rule-in**	0	0	8	0	3	49
**NRI**	0.127 (95% CI, 0.061-0.173)			0.068 (95% CI, 0.0-0.136)		
**NRI (dimensionless)**	0.195 (95% CI, 0.113-0.277); p value <0.001			IDI	0.065 (95% CI, 0.037-0.093)	

**Thresholds**	**Sensitivity** (95% CI)	**NPV** (95% CI)	**Specificity** (95% CI)	**PPV** (95% CI)
**hs-cTnT 5 ng/L**	100% (100-100%)	100% (100-100%)	28.8% (24.8-33.1%)	23.8% (19.4-27.8%)
**hs-cTnT 52 ng/L**	50.5% (41.3-60.4%)	89.9% (87.4-92.6%)	98.3% (97-99.3%)	86.9% (77.2-94.1%)
**cMyC 10 ng/L**	100% (100-100%)	100% (100-100%)	41.3% (36.8-45.9%)	27.3% (22.9-31.9%)
**cMyC 120 ng/L**	57.1% (47.5-67%)	91.1% (88.5-93.6%)	96.6% (94.8-98.1%)	78.9% (68.8-87.6%)

Legend: NRI = Net Reclassification Improvement; IDI = Integrated Discrimination Improvement; CI = Confidence Interval; NPV = Negative Predictive Value; PPV = Positive Predictive Value; AMI = Acute Myocardial Infarction, based on the adjudicated gold-standard diagnosis

Table S10. Derivation cohort – hs-cTnI

**Initial model**	New model - cMyC (10/120) – Derivation cohort					
**hs-cTnI**	No AMI (n=457)			AMI (n=96)		
	Rule-out	Observe	Rule-in	Rule-out	Observe	Rule-in
**Rule-out**	61	10	2	0	0	0
**Observe**	141	224	3	0	37	5
**Rule-in**	1	4	11	0	6	48
**NRI**	0.287 (95% CI, 0.217-0.336)			-0.010 (95% CI, -0.081-0.060)		
**NRI (dimensionless)**	0.276 (95% CI, 0.191-0.361); p value <0.001			IDI	0.090 (95% CI, 0.062-0.119)	

**Thresholds**	**Sensitivity** (95% CI)	**NPV** (95% CI)	**Specificity** (95% CI)	**PPV** (95% CI)
**hs-cTnI 2 ng/L**	100% (100-100%)	100% (100-100%)	15.9% (12.7-19.3%)	20% (16.3-23.8%)
**hs-cTnI 52 ng/L**	55.9% (46.2-66%)	91.3% (88.8-93.7%)	96.5% (94.9-98%)	77.3% (66.7-86.4%)
**cMyC 10 ng/L**	100% (100-100%)	100% (100-100%)	42.7% (38.3-47.4%)	26.9% (22.2-31.1%)
**cMyC 120 ng/L**	55.3% (45.8-64.8%)	91.2% (88.5-93.6%)	96.5% (94.8-98%)	77.1% (66.7-86.1%)

Legend: NRI = Net Reclassification Improvement; IDI = Integrated Discrimination Improvement; CI = Confidence Interval; NPV = Negative Predictive Value; PPV = Positive Predictive Value; AMI = Acute Myocardial Infarction, based on the adjudicated gold-standard diagnosis

Table S11. Net Reclassification Improvement – Onset of chest pain <3 hours prior to presentation

**Initial model**	New model - MyC (10/120) - chest pain for <3hrs					
**hs-cTnT**	No AMI (n=382)			AMI (n=78)		
	Rule-out	Observe	Rule-in	Rule-out	Observe	Rule-in
**Rule-out**	99	28	0	0	1	0
**Observe**	83	161	6	0	38	10
**Rule-in**	0	0	5	0	1	28
**NRI**	0.128 (95% CI, 0.055-0.181)			0.128 (95% CI, 0.044-0.213)		
**NRI (dimensionless)**	0.256 (95% CI, 0.157-0.356); p value <0.001			IDI	0.086 (95% CI, 0.052-0.119)	

**Thresholds**	**Sensitivity** (95% CI)	**NPV** (95% CI)	**Specificity** (95% CI)	**PPV** (95% CI)
**hs-cTnT 5 ng/L**	98.8% (95.8-100%)	99.2% (97.5-100%)	33.3% (28.6-38.1%)	23.3% (19-27.9%)
**hs-cTnT 52 ng/L**	37.2% (25.9-48.2%)	88.5% (85.3-91.4%)	98.7% (97.5-99.7%)	86.1% (73.3-96.9%)
**cMyC 10 ng/L**	100% (100-100%)	100% (100-100%)	46.4% (41.5-51.6%)	27.5% (22.3-32.5%)
**cMyC 120 ng/L**	49% (36.8-60%)	90.4% (87.4-93.2%)	97.2% (95.3-98.7%)	77.8% (65.2-89.7%)

**Initial model**	New model - MyC (10/120) - chest pain for <3hrs					
**hs-cTnI**	No AMI (n=381)			AMI (n=79)		
	Rule-out	Observe	Rule-in	Rule-out	Observe	Rule-in
**Rule-out**	76	11	1	0	0	0
**Observe**	109	169	4	0	39	8
**Rule-in**	0	5	6	0	4	28
**NRI**	0.257 (95% CI, 0.185-0.310)			0.051 (95% CI, -0.032-0.133)		
**NRI (dimensionless)**	0.308 (95% CI, 0.210-0.406); p value <0.001			IDI	0.101 (95% CI, 0.067-0.135)	

**Thresholds**	**Sensitivity** (95% CI)	**NPV** (95% CI)	**Specificity** (95% CI)	**PPV** (95% CI)
**hs-cTnI 2 ng/L**	100% (100-100%)	100% (100-100%)	23.2% (19-27.1%)	21.3% (16.8-25.7%)
**hs-cTnI 52 ng/L**	40.3% (29.5-51.2%)	88.7% (85.5-91.7%)	97.2% (95.5-98.7%)	74.8% (60.7-87.5%)
**cMyC 10 ng/L**	100% (100-100%)	100% (100-100%)	47.1% (42.4-52.2%)	28.2% (22.9-33.3%)
**cMyC 120 ng/L**	45.6% (34.7-57.3%)	89.6% (86.7-92.6%)	97.1% (95.3-98.7%)	76.6% (64.1-87.8%)

Legend: NRI = Net Reclassification Improvement; IDI = Integrated Discrimination Improvement; CI = Confidence Interval; NPV = Negative Predictive Value; PPV = Positive Predictive Value; AMI = Acute Myocardial Infarction, based on the adjudicated gold-standard diagnosis

Table S12. Net Reclassification Improvement – Onset of chest pain ≥3 hours prior to presentation

**Initial model**	**New model - MyC (10/120) - chest pain for ≥3hrs**					
**hs-cTnT**	No AMI (n=1172)			AMI (n=244)		
	Rule-out	Observe	Rule-in	Rule-out	Observe	Rule-in
**Rule-out**	255	77	0	0	0	0
**Observe**	202	569	34	1	69	24
**Rule-in**	0	7	28	0	11	139
**NRI**	0.084 (95% CI, 0.034-0.114)			0.049 (95% CI, 0.0-0.098)		
**NRI (dimensionless)**	0.133 (95% CI, 0.076-0.190); p value <0.001			IDI	0.044 (95% CI, 0.025-0.063)	

**Thresholds**	**Sensitivity** (95% CI)	**NPV** (95% CI)	**Specificity** (95% CI)	**PPV** (95% CI)
**hs-cTnT 5 ng/L**	100% (100-100%)	100% (100-100%)	28.4% (25.8-31%)	22.6% (20.1-25.1%)
**hs-cTnT 52 ng/L**	61.4% (55.6-67.3%)	92.3% (90.9-93.9%)	97% (96-97.9%)	81.1% (75.3-86.7%)
**cMyC 10 ng/L**	99.6% (98.7-100%)	99.8% (99.3-100%)	37.3% (34.8-40.3%)	24.9% (22-27.8%)
**cMyC 120 ng/L**	66.9% (61-72.6%)	93.2% (91.8-94.5%)	94.7% (93.4-96%)	72.5% (66.7-78.1%)

**Initial model**	New model - MyC (10/120) - chest pain for ≥3hrs					
**hs-cTnI**	No AMI (n=1156)			AMI (n=241)		
	Rule-out	Observe	Rule-in	Rule-out	Observe	Rule-in
**Rule-out**	152	31	1	0	0	0
**Observe**	305	581	21	1	61	16
**Rule-in**	1	24	40	0	18	145
**NRI**	0.240 (95% CI, 0.190-0.270)			-0.012 (95% CI, -0.061-0.036)		
**NRI (dimensionless)**	0.227 (95% CI, 0.170-0.285); p value <0.001			IDI	0.075 (95% CI, 0.056-0.094)	

**Thresholds**	**Sensitivity** (95% CI)	**NPV** (95% CI)	**Specificity** (95% CI)	**PPV** (95% CI)
**hs-cTnI 2 ng/L**	100% (100-100%)	100% (100-100%)	15.9% (14-18%)	19.9% (17.7-22%)
**hs-cTnI 52 ng/L**	67.5% (61.3-73.7%)	93.3% (91.9-94.7%)	94.4% (93.1-95.6%)	71.5% (65.4-77%)
**cMyC 10 ng/L**	99.6% (98.6-100%)	99.8% (99.3-100%)	38.1% (35.3-41%)	25.2% (22.4-28.1%)
**cMyC 120 ng/L**	66.9% (60.7-72.4%)	93.2% (91.7-94.6%)	94.6% (93.3-95.9%)	72.1% (66.2-78.3%)

Legend: NRI = Net Reclassification Improvement; IDI = Integrated Discrimination Improvement; CI = Confidence Interval; NPV = Negative Predictive Value; PPV = Positive Predictive Value; AMI = Acute Myocardial Infarction, based on the adjudicated gold-standard diagnosis

Table S13. Specificity of biomarkers at presentation for adjudicated diagnosis of Acute Myocardial Infarction at the 99th centile

	cMyC at 87 ng/L	***hs-cTnI at 26 ng/L***	***hs-cTnT at 14 ng/L***
Sensitivity	69.6% (95% CI, 64.9-74.2%)	70.6% (95% CI, 65.6-75.5%)	91% (95% CI, 87.8-94.1%)
Specificity	93% (95% CI, 91.7-94.3%)	92.3% (95% CI, 90.8-93.5%)	76.4% (95% CI, 74.3-78.6%)
NPV	93.6% (95% CI, 92.3-94.7%)	93.8% (95% CI, 92.5-94.9%)	97.6% (95% CI, 96.7-98.4%)
PPV	67.7% (95% CI, 62.9-72.4%)	65.4% (95% CI, 60.2-70.6%)	44.4% (95% CI, 40.7-48.2%)

Table S14. Prognosis – Harrell's C and Somers' D statistics

n=1876	**cMyC**	**hs-cTnT**	**p value***	**est.cov**
FU AMI				
Harrell's C Statistic	0.725	0.706	0.251	0.000
Somers' D ± SD	0.450 ±0.045	0.411 ±0.048		
FU death				
Harrell's C Statistic	0.765	0.782	0.142	0.000
Somers' D ± SD	0.530 ±0.034	0.564 ±0.031		
FU composite EP				
Harrell's C Statistic	0.745	0.749	0.667	0.000
Somers' D ± SD	0.489 ±0.029	0.498 ±0.029		
n=1857	**cMyC**	**hs-cTnI**	**p value**	**est.cov**
FU AMI				
Harrell's C Statistic	0.724	0.714	0.577	0.000
Somers' D ± SD	0.447 ±0.047	0.429 ±0.047		
FU death				
Harrell's C Statistic	0.767	0.732	0.001	0.000
Somers' D ± SD	0.535 ±0.034	0.464 ±0.036		
FU composite EP				
Harrell's C Statistic	0.746	0.722	0.008	0.000
Somers' D ± SD	0.492 ±0.029	0.443 ±0.030		
n=1774	**cMyC**	**s-cTnI**	**p value**	**est.cov**
FU AMI				
Harrell's C Statistic	0.719	0.504	<0.001	0.000
Somers' D ± SD	0.438 ±0.047	0.007 ±0.002		
FU death				
Harrell's C Statistic	0.763	0.507	<0.001	0.000
Somers' D ± SD	0.527 ±0.035	0.014 ±0.011		
FU composite EP				
Harrell's C Statistic	0.741	0.503	<0.001	0.000
Somers' D ± SD	0.483 ±0.030	0.007 ±0.008		

Legend: FU = Follow-up event, AMI = Acute Myocardial Infarction (based on the adjudicated gold-standard diagnosis), composite EP = endpoint combining death and AMI during FU (excluding index event), Somers' D quoted ± SD = Standard error of Somers' D, est.cov = estimated covariance between two C indices; *p value for direct comparison between biomarkers

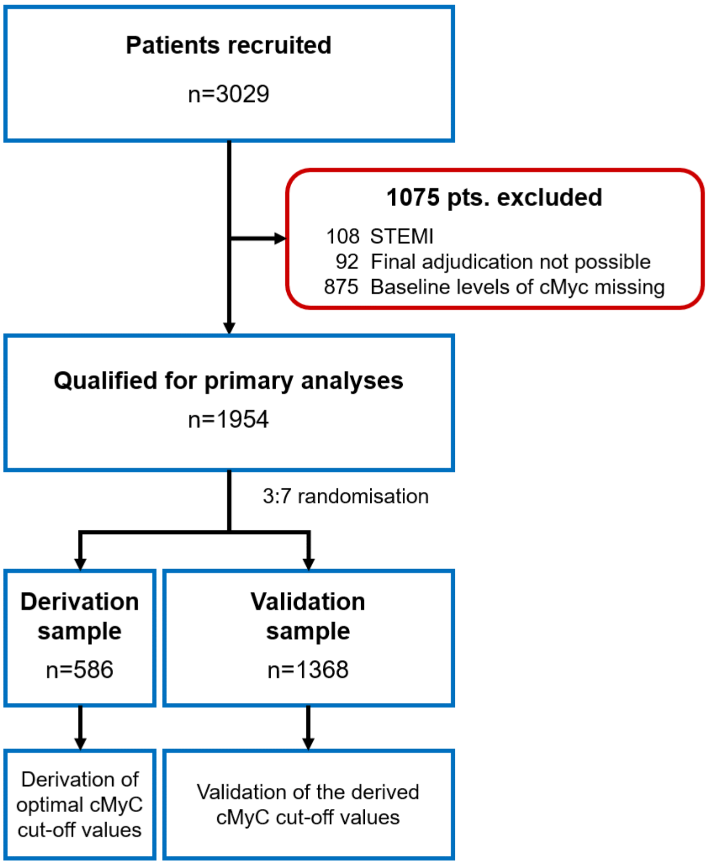

Figure S1

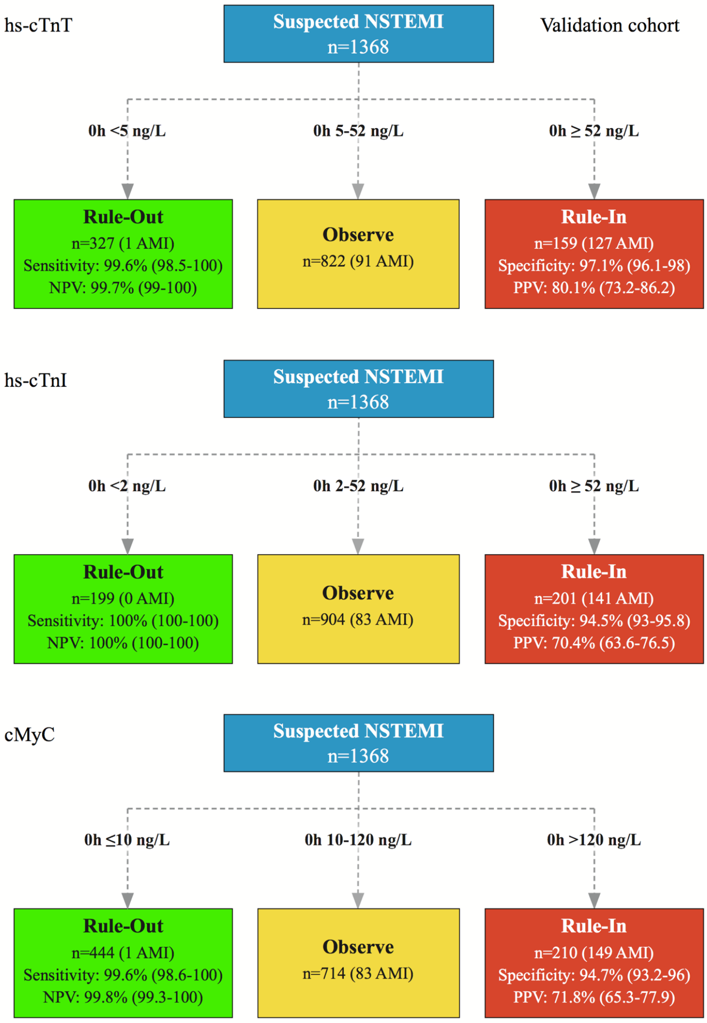

Figure S2

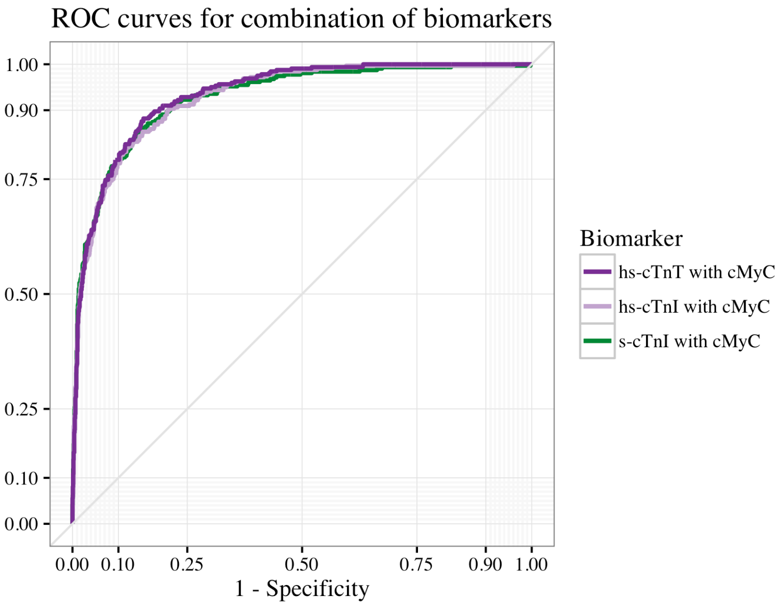

Figure S3

Survival curve for hs-cTnT - 3 year follow-up

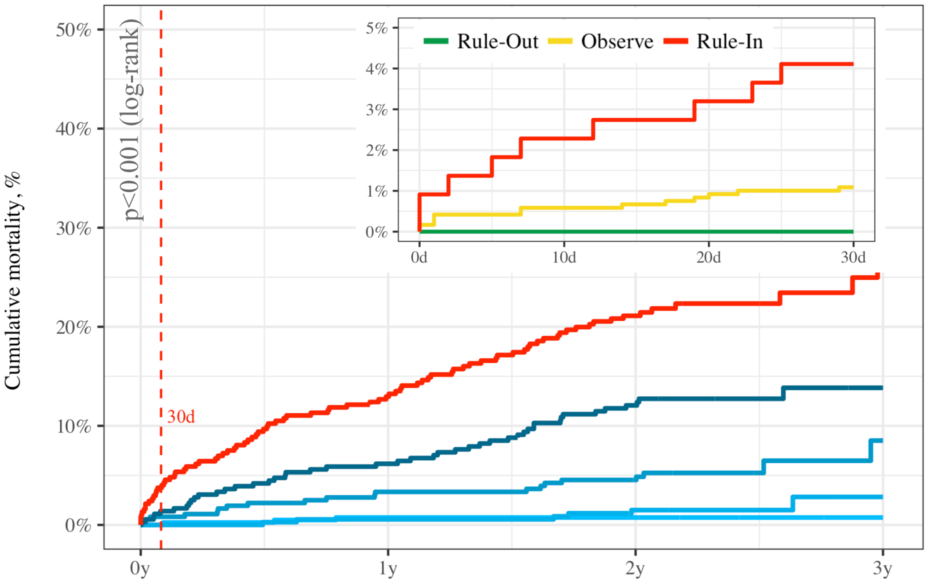

Number at risk at 30d

	10d	20d	30d
Rule-Out	457	457	456
Observe	1194	1187	1183
Rule-In	217	214	212

Number at risk at 3 years

	1y	2y	3y
1st quintile	407	391	351
2nd quintile	367	352	300
3rd quintile	364	343	296
4th quintile	359	329	275
5th quintile	371	318	259

Survival curve for hs-cTnI - 3 year follow-up

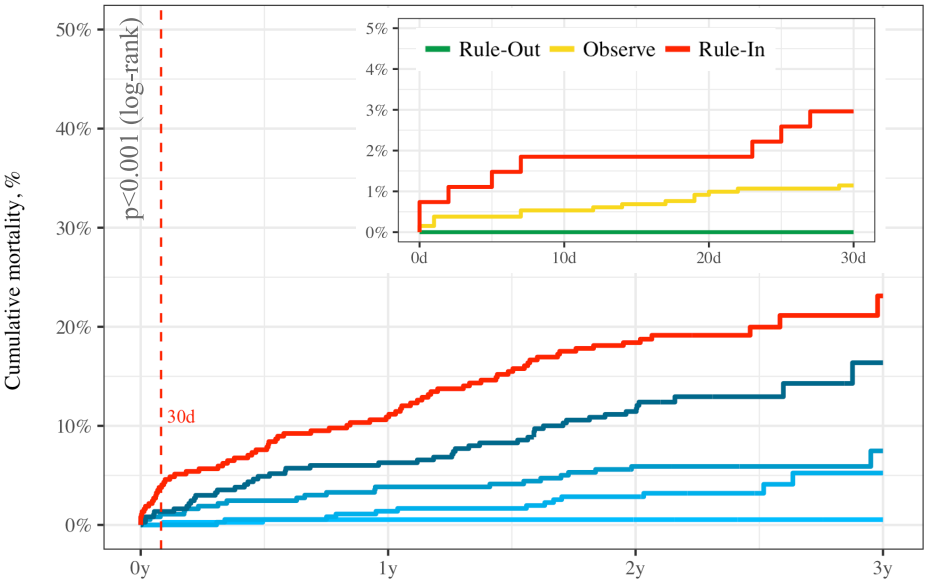

Number at risk at 30d

	10d	20d	30d
Rule-Out	271	271	271
Observe	1309	1303	1296
Rule-In	269	265	265

Number at risk at 3 years

	1y	2y	3y
1st quintile	379	367	315
2nd quintile	366	354	315
3rd quintile	368	344	299
4th quintile	368	340	288
5th quintile	368	319	261

Survival curve for cMyC - 3 year follow-up

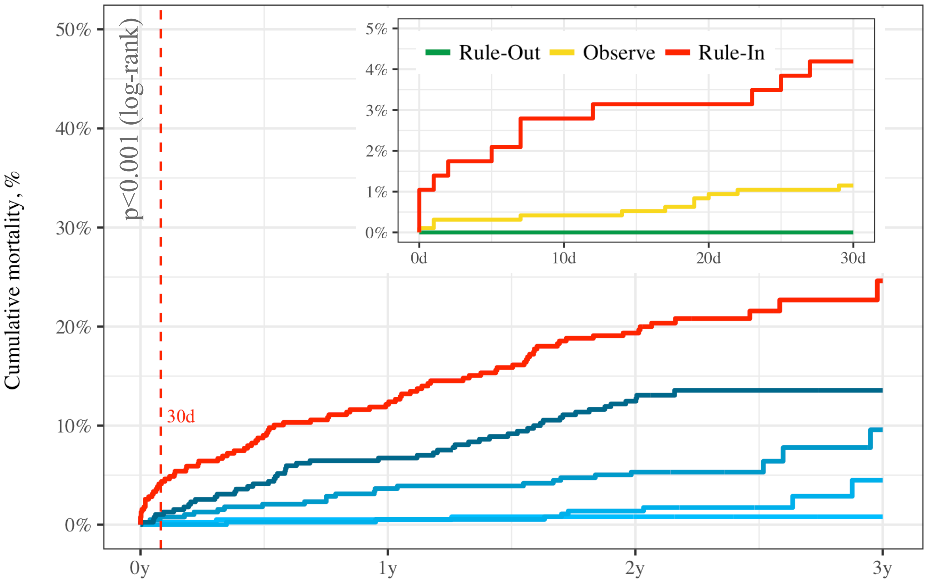

Number at risk at 30d

	10d	20d	30d
Rule-Out	706	705	704
Observe	956	953	948
Rule-In	284	278	277

Number at risk at 3 years

	1y	2y	3y
1st quintile	390	377	327
2nd quintile	388	374	324
3rd quintile	390	365	320
4th quintile	391	356	297
5th quintile	387	335	283

Figure S4A-C 1st quintile 2nd quintile 3rd quintile 4th quintile 5th quintile

Supplemental figure legends:

Figure S1: Flowchart outlining recruitment numbers and exclusions from test cohort

Figure S2: Flow of participants, depending on each biomarker used, according to ESC guideline[6] for hs-cTnT and hs-cTnI, and theoretical model for the novel biomarker cMyC; AMI = Acute Myocardial Infarction, based on the adjudicated gold-standard diagnosis

Figure S3: ROC curves describing the diagnostic performance of the combination of cMyC with hs-cTnT (dark purple line; AUC 0.935*), hs-cTnI (light purple line; AUC 0.929) and s-cTnI (green line; AUC 0.928*); *$p<0.05$

Figure S4: Cumulative incidence of death in all patients based on biomarker value at presentation: all-comers underwent follow-up for up to 3 years. Survival curves are plotted for hs-cTnT, hs-cTnI and cMyC based on quintiles for a three year follow-up, and separated in risk groups 'Rule-Out', 'Observe' and 'Rule-In'[6] at 30 day follow-up. Amongst quintiles, the HR for hs-cTnT at three year follow-up was 2.3 (95% CI, 0.6-9.0) in the second quintile, 7.7 (95% CI, 2.3-25.8) in the third, 17.7 (95% CI, 5.5-57.1) in the fourth and 33.6 (95% CI, 10.6-106.3) in the fifth quintile. The HR for hs-cTnI was 6.6 (95% CI, 1.5-29.2), 11.3 (95% CI, 2.7-48.3), 25.1 (95% CI, 6.1-103.3) and 39.7 (95% CI, 9.7-161.8), respectively. The HR for cMyC was 2.6 (95% CI, 0.7-10.0), 7.8 (95% CI, 2.3-25.9), 17.2 (95% CI, 5.4-55.0) and 29.4 (95% CI, 9.3-93.2).

The quintiles comprise of the following tiers:

hs-cTnT (figure S3a): [0.0, 4.1) [4.1, 7.1) [7.1, 12.1) [12.1, 27.5) [27.5, 1750.0]

hs-cTnI (figure S3b): [0.2, 2.3) [2.3, 3.6) [3.6, 6.8) [6.8, 22.9) [22.9, 25351.6]

cMyC (figure S3c): [1.27, 6.92) [6.92, 12.24) [12.24, 24.19) [24.19, 71.71) [71.71, 4369.03]

Additional HTML-document:

Interactive_ROC.html: This represents an interactive document allowing the reader to assess different cut-offs of the respective biomarkers in isolation as well as in comparison to cMyC. This can be further adjusted to match publishing guidelines if the journal wishes to include in the web supplement.

Supplemental References

1. Roffi M, Patrono C, Collet J-P, Mueller C, Valgimigli M, Andreotti F, Bax JJ, Borger MA, Brotons C, Chew DP, Gencer B, Hasenfuss G, Kjeldsen K, Lancellotti P, Landmesser U, Mehilli J, Mukherjee D, Storey RF, Windecker S, Baumgartner H, Gaemperli O, Achenbach S, Agewall S, Badimon L, Baigent C, Bueno H, Bugiardini R, Carerj S, Casselman F, Cuisset T, Erol Ç, Fitzsimons D, Halle M, Hamm C, Hildick-Smith D, Huber K, Iliodromitis E, James S, Lewis BS, Lip GYH, Piepoli MF, Richter D, Rosemann T, Sechtem U, Steg PG, Vrints C, Luis Zamorano J, Management of Acute Coronary Syndromes in Patients Presenting without Persistent ST-Segment Elevation of the European Society of Cardiology. 2015 ESC Guidelines for the management of acute coronary syndromes in patients presenting without persistent ST-segment elevation: Task Force for the Management of Acute Coronary Syndromes in Patients Presenting without Persistent ST-Segment Elevation of the European Society of Cardiology (ESC). *Eur Heart J*. 2016;37:267–315.

2. Thygesen K, Alpert JS, Jaffe AS, Simoons ML, Chaitman BR, White HD, Thygesen K, Alpert JS, White HD, Jaffe AS, Katus HA, Apple FS, Lindahl B, Morrow DA, Chaitman BR, Clemmensen PM, Johanson P, Hod H, Underwood R, Bax JJ, Bonow RO, Pinto F, Gibbons RJ, Fox KA, Atar D, Newby LK, Galvani M, Hamm CW, Uretsky BF, Steg PG, Wijns W, Bassand J-P, Menasche P, Ravkilde J, Ohman EM, Antman EM, Wallentin LC, Armstrong PW, Simoons ML, Januzzi JL, Nieminen MS, Gheorghiade M, Filippatos G, Luepker RV, Fortmann SP, Rosamond WD, Levy D, Wood D, Smith SC, Hu D, Lopez-Sendon J-L, Robertson RM, Weaver D, Tendera M, Bove AA, Parkhomenko AN, Vasilieva EJ, Mendis S. Third universal definition of myocardial infarction. *J Am Coll Cardiol*. 2012;60:1581–1598.

3. Thygesen K, Alpert JS, White HD, Joint ESC/ACCF/AHA/WHF Task Force for the Redefinition of Myocardial Infarction. Universal definition of myocardial infarction. *Eur Heart J*. 2007;28:2525–2538.

4. Thygesen K, Mair J, Giannitsis E, Mueller C, Lindahl B, Blankenberg S, Huber K, Plebani M, Biasucci LM, Tubaro M, Collinson P, Venge P, Hasin Y, Galvani M, Koenig W, Hamm C, Alpert JS, Katus H, Jaffe AS, Study Group on Biomarkers in Cardiology of ESC Working Group on Acute Cardiac Care. How to use high-sensitivity cardiac troponins in acute cardiac care. *Eur Heart J*. 2012;33:2252–2257.

5. Apple FS, Jesse RL, Newby LK, Wu AHB, Christenson RH, Cannon CP, Francis G, Christenson RH, Morrow DA, Ravkilde J, Apple FS, Storrow AB, Tang W, IFCC Comittee on Standardization of Markers of Cardiac Damege, Jaffe AS, Mair J, Newby LK, Ordonez-Llanos J, Pagani F, Panteghini M, Tate J, Wu AHB, National Academy of Clinical Biochemistry. National Academy of Clinical Biochemistry and IFCC Committee for Standardization of Markers of Cardiac Damage Laboratory Medicine Practice Guidelines: analytical issues for biochemical markers of acute coronary syndromes. *Clin Chem*. 2007;53:547–551.

6. Hamm CW, Bassand J-P, Agewall S, Bax J, Boersma E, Bueno H, Caso P, Dudek D, Gielen S, Huber K, Ohman M, Petrie MC, Sonntag F, Uva MS, Storey RF, Wijns W, Zahger D, ESC Committee for Practice Guidelines. ESC Guidelines for the management of acute coronary syndromes in patients presenting without persistent ST-segment elevation: The Task Force for the management of acute coronary syndromes (ACS) in patients presenting without persistent ST-segment elevation of the European Society of Cardiology (ESC). *Eur Heart J*. 2011;32:2999–3054.

7. Reichlin T, Irfan A, Twerenbold R, Reiter M, Hochholzer W, Burkhalter H, Bassetti S, Steuer S, Winkler K, Peter F, Meissner J, Haaf P, Potocki M, Drexler B, Osswald S, Mueller C. Utility of Absolute and Relative Changes in Cardiac Troponin Concentrations in the Early Diagnosis of Acute Myocardial Infarction. *Circulation*. 2011;124:136–145.

8. Irfan A, Reichlin T, Twerenbold R, Meister M, Moehring B, Wildi K, Bassetti S, Zellweger C, Gimenez MR, Hoeller R, Murray K, Sou SM, Mueller M, Mosimann T, Reiter M, Haaf P, Ziller R, Freidank H, Osswald S, Mueller C. Early diagnosis of myocardial infarction using absolute and relative changes in cardiac troponin concentrations. *Am J Med*. 2013;126:781–788.e2.

9. Biener M, Mueller M, Vafaie M, Keller T, Blankenberg S, White HD, Katus HA, Giannitsis E. Comparison of a 3-hour versus a 6-hour sampling-protocol using high-sensitivity cardiac troponin T for rule-out and rule-in of non-STEMI in an unselected emergency department population. *Int J Cardiol*. 2013;167:1134–1140.

10. Wildi K, Reichlin T, Twerenbold R, Mäder F, Zellweger C, Moehring B, Stallone F, Minners J, Gimenez MR, Hoeller R, Murray K, Sou SM, Mueller M, Denhaerynck K, Mosimann T, Reiter M, Haaf P, Meller B, Freidank

H, Osswald S, Mueller C. Serial changes in high-sensitivity cardiac troponin I in the early diagnosis of acute myocardial infarction. *Int J Cardiol*. 2013;168:4103–4110.

11. Biener M, Giannitsis E, Lamerz J, Mueller-Hennessen M, Vafaie M, Katus HA. Prognostic value of elevated high-sensitivity cardiac troponin T levels in a low risk outpatient population with cardiovascular disease. *Eur Heart J Acute Cardiovasc Care*. 2016;5:409–418.

12. Mueller M, Biener M, Vafaie M, Doerr S, Keller T, Blankenberg S, Katus HA, Giannitsis E. Absolute and relative kinetic changes of high-sensitivity cardiac troponin T in acute coronary syndrome and in patients with increased troponin in the absence of acute coronary syndrome. *Clin Chem*. 2012;58:209–218.

13. Vasile VC, Saenger AK, Kroning JM, Jaffe AS. Biological and Analytical Variability of a Novel High-Sensitivity Cardiac Troponin T Assay. *Clin Chem*. 2010;56:1086–1090.

14. Wu AHB, Lu QA, Todd J, Moecks J, Wians F. Short- and Long-Term Biological Variation in Cardiac Troponin I Measured with a High-Sensitivity Assay: Implications for Clinical Practice. *Clin Chem*. 2008;55:52–58.

15. Hammarsten O, Fu MLX, Sigurjonsdottir R, Petzold M, Said L, Landin-Wilhelmsen K, Widgren B, Larsson M, Johanson P. Troponin T Percentiles from a Random Population Sample, Emergency Room Patients and Patients with Myocardial Infarction. *Clin Chem*. 2012;58:628–637.

16. Reichlin T, Irfan A, Twerenbold R, Reiter M, Hochholzer W, Burkhalter H, Bassetti S, Steuer S, Winkler K, Peter F, Meissner J, Haaf P, Potocki M, Drexler B, Osswald S, Mueller C. Utility of Absolute and Relative Changes in Cardiac Troponin Concentrations in the Early Diagnosis of Acute Myocardial Infarction. *Circulation*. 2011;124:136–145.

17. Giannitsis E, Kurz K, Hallermayer K, Jarausch J, Jaffe AS, Katus HA. Analytical validation of a high-sensitivity cardiac troponin T assay. *Clin Chem*. 2010;56:254–261.

18. Apple FS, Jaffe AS. Clinical implications of a recent adjustment to the high-sensitivity cardiac troponin T assay: user beware. *Clin Chem*. 2012;58:1599–1600.

19. Kuster N, Dupuy A-M, Monnier K, Baptista G, Bargnoux A-S, Badiou S, Jeandel C, Cristol J-P. Implications of adjustment of high-sensitivity cardiac troponin T assay. *Clin Chem*. 2013;59:570–572.

20. Hallermayer K, Jarausch J, Menassanch-Volker S, Zaugg C, Ziegler A. Implications of adjustment of high-sensitivity cardiac troponin T assay. *Clin Chem*. 2013;59:572–574.

21. Kavsak PA, Hill SA, McQueen MJ, Devereaux PJ. Implications of adjustment of high-sensitivity cardiac troponin T assay. *Clin Chem*. 2013;59:574–576.

22. Wildi K, Twerenbold R, Jaeger C, Rubini Giménez M, Reichlin T, Stoll M, Hillinger P, Puelacher C, Boeddinghaus J, Nestelberger T, Grimm K, Grob M, Rentsch K, Arnold C, Mueller C. Clinical impact of the 2010-2012 low-end shift of high-sensitivity cardiac troponin T. *Eur Heart J Acute Cardiovasc Care*. 2016;5:399–408.

23. Koerbin G, Tate J, Potter JM, Cavanaugh J, Glasgow N, Hickman PE. Characterisation of a highly sensitive troponin I assay and its application to a cardio-healthy population. *Clin Chem Lab Med*. 2012;50:1–8.

24. Apple FS, Smith SW, Pearce LA, Ler R, Murakami MM. Use of the Centaur TnI-Ultra assay for detection of myocardial infarction and adverse events in patients presenting with symptoms suggestive of acute coronary syndrome. *Clin Chem*. 2008;54:723–728.

25. Melanson SEF, Morrow DA, Jarolim P. Earlier Detection of Myocardial Injury in a Preliminary Evaluation Using a New Troponin I Assay With Improved Sensitivity. *Am J Clin Pathol*. 2007;128:282–286.

26. Levey AS, Coresh J, Greene T, Stevens LA, Zhang YL, Hendriksen S, Kusek JW, Van Lente F, Chronic Kidney Disease Epidemiology Collaboration. Using standardized serum creatinine values in the modification of diet in renal disease study equation for estimating glomerular filtration rate. *Ann Intern Med*. 2006;145:247–254.

27. Baker JO, Tyther R, Liebetrau C, Clark J, Howarth R, Patterson T, Möllmann H, Nef H, Sicard P, Kailey B, Devaraj R, Redwood SR, Kunst G, Weber E, Marber MS. Cardiac myosin-binding protein C: a potential early biomarker of myocardial injury. *Basic Res Cardiol*. 2015;110:23.
